# Vertex models capturing subcellular scales in epithelial tissues

**DOI:** 10.1371/journal.pcbi.1012993

**Published:** 2025-05-21

**Authors:** Zoë Lange, Franziska Matthäus, Mingfeng Qiu

**Affiliations:** 1 Frankfurt Institute for Advanced Studies, Frankfurt am Main, Germany; 2 Department of Physics, Goethe-Universität Frankfurt am Main, Frankfurt am Main, Germany; 3 Department of Computer Science and Mathematics, Goethe-Universität Frankfurt am Main, Frankfurt am Main, Germany; 4 Laboratoire de Physique de l’École Normale Supérieure, CNRS, ENS, Université PSL, Sorbonne Université, Université Paris Cité, Paris, France; 5 School of Mathematics and Statistics, University of Canterbury, Christchurch, New Zealand; Collège de France, FRANCE

## Abstract

Vertex models provide a robust theoretical framework for studying epithelial tissues as a network of cell boundaries. They have been pivotal in exploring properties such as cell packing geometry and rigidity transitions. Recently, extended vertex models have become instrumental in bridging the subcellular scales to the tissue scale. Here, we review extensions of the model aiming to capture experimentally observed subcellular features of epithelial tissues including heterogeneity in myosin activity across the tissue, non-uniform contractility structures, and mechanosensitive feedback loops. We discuss how these extensions change and challenge current perspectives on observables of macroscopic tissue properties. First, we find that extensions to the vertex model can change model properties significantly, impacting the critical threshold and in some cases even the existence of a rigidity transition. Second, we find that packing disorder can be explained by models employing different subcellular mechanisms, indicating a source of stochasticity and gradual local size changes as common mesoscopic motifs in the mechanics of tissue organization. We address complementary models and statistical inference, putting vertex models in a broader methodological context and we give a brief overview of software packages utilized in increasingly complex vertex model studies. Our review emphasizes the need for more comparative, systematic studies that identify specific classes of vertex models which share a set of well-defined properties, as well as a more in-depth discussion of modeling choices and their biological motivations.

## Author summary

The vertex model is a theoretical and computational framework to study skin-like tissues by representing them as networks of cell boundaries. In this article, we review the latest advances in the design and application of vertex models. We focus on how the model framework is extended to capture refined structures, dynamics, and feedback within individual cells, and how the extensions affect tissue properties such as stiffness and cell packing. Based on the surveyed results we discuss future directions of research in this field. We also compare vertex models with related tissue models, talk about methods to estimate mechanical forces from experimental data by leveraging the same vertex-edge network, and recommend software for vertex model simulations. This review helps researchers choose the right version of the vertex model for their biological question and encourages collaboration among biological physicists and mathematical biologists to better understand the mechanisms underlying observed behavior and organization of tissues in animals.

## 1 Introduction

Tissues are basic forms of biological materials and constitute the organizational level between individual cells and entire organs. A typical tissue is the epithelium, a layer of cells lining the surfaces of animal organs. Epithelial cells are polarized along the apicobasal axis [1]. They bear locally specific proteins both on the surface and internally. At the basal surface, each cell is decorated with integrins, a family of specialized adhesive trans-membrane proteins. Integrins attach the cell to the basal lamina, a dense polymer substrate, which is a type of extracellular matrix (ECM) [2,3]. The apical surface, which can be associated with an apical ECM, is either in contact with the basal surface of another layer or forms an interface with a lumen or air external to the organ [4]. Confluence of the tissue is mostly ensured through adhesive trans-membrane protein complexes of the cadherin family in the apical region. They form cell-cell junctions such that cells are tightly packed together to form a monolayer. The shape and motion of biological tissues emerge from the underlying cellular structures and subcellular activities, resulting in more complicated dynamics than observed in passive materials [5–8]. Understanding the behavior of cells and tissues, such as how their size and shape are controlled by their subcellular activities and how large-scale events like convergent extension are coordinated, is central to the study of development, homeostasis and disease [9–16].

The vertex model is a successful theoretical framework to study epithelial tissue morphology at cellular resolution. It originates from a geometric description of cellular packings [17–19]. Inspired by a vertex-based study of dynamics of cellular patterns in soap foams [20], the epithelial model was later augmented with a mechanical description [21–23]. The vertex model abstracts an epithelial tissue as a tiling of polyhedrons [24–27]. A common dimensional reduction approach is a representation of the tissue in two dimensions (2D) where cells are reduced to polygons in an apical cross-section [23,27,28]. In the resulting network, edges represent junctions between neighboring cells and vertices correspond to sites where three or more cells meet. The polygonal configuration is commonly governed by a phenomenological Hamiltonian, a function describing the energy of the system dependent on the geometry of individual cells. The energy function in the seminal model by Farhadifar et al. [23] represents three major force contributions in the cell: the contractile forces generated by the cytoskeleton when myosin motor proteins walk on actin filaments, the resistance from the incompressible cytosolic fluid and the adhesion between neighboring cells. In addition, this framework also includes a set of update rules that account for topological changes in the network that are necessary to perform cell division, cell extrusion, and cell neighbor exchanges. Vertex models like the one of Farhadifar et al. [23] offer a theoretical foundation to bridge subcellular activities, in [23] actomyosin contractility and a cell cycle, to tissue-scale behavior. The vertex-based framework provides an intuitive discretization of a confluent tissue on a subcellular level. Vertex models have been successful in exploring the properties of epithelial tissues. They are able to relate cell packing geometry to the balance of physical forces and to predict a rigidity transition of the tissue in response to changes in single-cell properties [23,29–31]. Vertex models have also been applied to model a variety of specific morphogenetic processes, e.g., *Drosophila* germband extension [32,33], ventral furrow formation [34], dorsal closure [35,36], and wound healing [37]. Two seminal reviews have been dedicated to the vertex model [27,28]: The first gives a detailed overview of the formulation first introduced in [23] and its applications by a wide community of modelers [28]. A second review presents a general form of the model in the language of virtual work and classifies different vertex models based on the spatial dimensions and geometry that they cover [27]. Both reviews have also highlighted extensions to the vertex model, such as incorporating planar cell polarity and the generalization to a three-dimensional (3D) geometry. A recent book has covered the vertex model, focusing on a didactic presentation of the construction of the model and earlier developments in the field [38].

In recent years, extensions and modifications of the vertex model, specifically aiming to capture subcellular structures, dynamics, and feedback mechanisms, are being explored, highlighting the need for a coherent picture of model variants and their respective properties. By now, the vertex model has been established as one of the most popular models to study epithelial tissues from a physical perspective, with an increasingly large and interdisciplinary community. The vertex model introduced by Farhadifar et al. [23], popularized by its capability to capture a solid-to-fluid transition in tissues [29,39], is by far the most cited vertex model study and recently being referred to as the “conventional model” or “standard model” in the realm of vertex models [40–42]; it serves as a *reference model* in this review. Meanwhile, alternative and extended versions of the model have been developed, partly driven by advances in experimental tools like genetic engineering and imaging techniques. Novel details on the motor proteins and related molecular processes at the nano- and micro-scale are being discovered, which the reference model does not capture. For example, while the reference model only implements uniform contractility along the cell perimeter, during development, actomyosin is first localized in the medial region before later on localizing to the cortex along cell-cell junctions [43]. To account for these experimental observations, a large number of variants have been proposed to extend the vertex model, ranging from stochastic parameters in the energy function to coupling with biochemical networks, e.g., [34,36,44,45]. However, recent studies do not always explore physical properties and compare new model variants to properties of the reference model, and it remains unclear what impact extensions have on the theory of vertex models.

Here, we review novel extensions of the vertex model in the context of their motivations and discuss insights on the fundamental properties of vertex models obtained by these extensions. We build on frameworks set by previous works and reviews and categorize model extensions that address different complexity limits of the vertex model. Our objective consists of three specific goals: First, we aim to survey the latest model extensions and to provide a topical reference for vertex model practitioners to make informed modeling choices, when studying specific biological and physical questions, or for developing further extensions. Second, we strive to make this review an accessible and up-to-date introduction for researchers new to this field, including both theoreticians and experimentalists. Finally, we summarize key questions to be studied and debated. We find that a perspective attracting increasing attention is the notion of bridging spatial scales: coupling the vertex model to subcellular activities and studying emergent behavior on the tissue scale. Incorporating active regulation at smaller scales into a scalable model framework provides many opportunities and challenges for future studies. In structuring our review, we start by introducing what we consider our reference model followed by extensions to the framework. In each section, we highlight experimental observations and theoretical considerations supporting a particular extension, describe the mathematical formulation, and give examples of applications. Specifically, we cover non-uniform and adaptive contractility, coupling with cell-cell junctional remodeling mechanisms related to mechanosensitivity, active topological transitions, and tissue rheology. Then we examine how various model extensions preserve or modify tissue properties, the most well-known of which are tissue rigidity and cell packing geometry. We highlight the lack of comparative studies and unifying frameworks in this aspect. Finally, we put the model in the broader context of complementary models and methods to study tissue behavior. As extensions with a subcellular focus have been mostly implemented in 2D apical vertex models so far, we will restrict our introduction of the model formalism to 2D and only explicitly highlight three-dimensional aspects if they are relevant to the implementation or conclusions regarding subcellular aspects.

## 2 A vertex model for epithelial tissues

**Fig 1:**
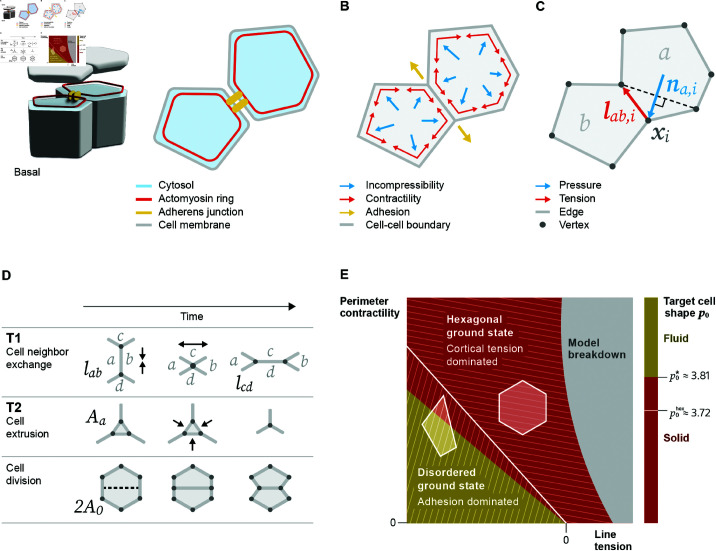
Forces, topological transitions, and phase transitions in a vertex model for confluent epithelial tissues. (A) The vertex model incorporates what are considered the main mechanical components in polarized epithelial cells: cytosol filling the volume of a cell; in the apical region: an actomyosin ring along the cell perimeter and adherens junctions connecting two cells, and a cell membrane enclosing the volume. The two-dimensional model is a cross-section close to the apical surface of the tissue. (B) Cells in the tissue are approximated by polygons that form a confluent tiling. In the vertex model, we attribute specific forces to the mechanical components: The cytosol resists compression and expansion forces; the actomyosin ring exerts contractile forces on a cell and the adherens junctions result in attractive forces along the cell-cell boundary. The adhesion forces compete with the cortical tension from the cell membrane and actomyosin ring. (C) Vertices in the network satisfy force balance based on the forces from the mechanical components. Effective forces on a vertex *i* are along the edge length vector ${𝐥}_{ab,i}$ pointing away from reference vertex *i* (tension) and the normal vector ${𝐧}_{a,i}$ defined as perpendicular to the line connecting the two neighbors of vertex *i* and pointing towards the outside of the cell (pressure). (D) A common set of update rules accounts for topological transitions in the temporal evolution of a vertex network. T1 transitions are cell neighbor exchanges where an edge between cells *a* and *b* falls below a certain threshold and continues to shrink until it vanishes; then, a new edge between cells *c* and *d* is inserted perpendicular to the old edge. T2 transitions are cell extrusions where a cell with an area below a threshold value vanishes and leaves a single vertex. Cell division, as part of proliferation, occurs when a cell splits into two. (E) Phase diagram of tissue mechanical states as a function of parameters *k*_*P*_ (perimeter contractility) and *k*_*l*_ (line tension). When the control parameter $p_{0}=P_{0}/\sqrt{A_{0}}$ is below $p_{0}^{hex}\approx 3.72$ (white line), the ground state is a regular hexagonal lattice, which becomes unstable once *p*_0_ increases past $p_{0}^{hex}$ [23,39]. For a disordered, metastable tissue, a solid-to-fluid transition occurs as *p*_0_ increases past $p_{0}^{*}\approx 3.81$, characterized by vanishing energy barriers for T1 transitions [29]. The exact threshold of the solid-to-fluid transition can vary depending on cell packing disorder [10] and implementation details of the dynamics [46,47]. The gray area marks where the model breaks down due to vanishing areas.

In preparation for our review, we briefly recall the formulation and properties of the vertex model that the reviewed extensions are based on [23,29,39]. In a confluent cellular tissue such as an epithelial monolayer, cells are bound together through adherens junctions – adhesive molecular complexes at cell-cell boundaries. Tension can be generated by the contractile actomyosin cytoskeleton along the perimeters of the cells and propagated through the adherens junctions. Both the actomyosin ring and the adherens junctions are located close to the apical cortices of the cells. This allows, in many cases, a description of a confluent tissue as a 2D polygonal network, with each edge representing a cell-cell junction consisting of the adherens junctions, the cell-cell boundary, and the actomyosin cytoskeleton on both sides of the boundary (Fig 1A and 1B). Vertices represent junctions where three or more cells meet, and their coordinates, the degrees of freedom of the model, are subject to the physical law of force balance (Fig 1B and 1C). In a vertex model simulation, one usually proceeds by iterating between two steps: (1) update vertex positions based on the forces exerted by the neighboring cells (1C); (2) perform topological transitions in the vertex network that allow for cell neighbor exchanges, cell extrusions, and cell divisions based on a set of rules (Fig 1D). In the following, we will introduce the two steps specifically for the reference model [23,39] and we will briefly discuss the resulting properties of the model [29].

First, we introduce the equations of motion that describe the dynamics of the vertices of a network of epithelial cells. As the considered tissue motion is typically in a regime where viscous forces dominate inertial forces, it is assumed that the system is governed by over-damped dynamics:

$$\lambda \;\frac{d{𝐱}_{i}}{dt}={𝐅}_{i}.$$ (1)

Here, the left-hand side is a friction force with vertex velocity derived from vertex position ${𝐱}_{i}$. λ is an effective drag coefficient that accounts for dissipation from external sources. External dissipation arises from interactions with the environment, e.g., the ECM polymer substrate. While external friction is commonly used to model system friction and internal dissipation is neglected, more general formulations are possible. We discuss dissipation and its effect on time scales and how recent models implement this in Sect 4.

The friction force balances ${𝐅}_{i}$, the sum of all other forces exerted on vertex *i*. Two approaches have been used to formulate the force equations driving vertex motion. One proposes explicit functional forms of the forces by considering the membrane tension and the cortical pressure directly [21]. A more popular approach involves specifying a phenomenological Hamiltonian to derive ${𝐅}_{i}$ [22,23]. While this phenomenological Hamiltonian is generally not a conserved total energy, it has proven useful in describing tissue mechanics in two ways. First, it is well documented that the ground states of the phenomenological Hamiltonian, i.e., the most relaxed configurations of the network, display fundamental properties also observed in tissues (Fig 1E) [23,29,39], from which the vertex model’s popularity today likely arises. Second, the phenomenological Hamiltonian serves as a mathematical tool to derive mechanical forces, providing an extendable framework for modeling realistic tissue morphologies [22,27,28]. Compared to the force-based formulations of the vertex model, the derivative of the phenomenological Hamiltonian results in qualitatively similar forces but is not strictly equivalent [28]. For our review, we will follow the approach based on the phenomenological Hamiltonian due to its more widespread use. We will refer to the phenomenological Hamiltonian as an *energy function*, a terminology widely adopted in the field to emphasize the analogy to energy in passive systems [27,28].

Let E(𝐱) be the energy of the system dependent on all vertex positions **x**. Minimizing *E* with respect to the coordinates of vertex *i*, we can rewrite the equations of motion as

$$\lambda \;\frac{d{𝐱}_{i}}{dt}=-\nabla_{i}\;E(𝐱),$$ (2)

where $\nabla_{i}$ denotes the gradient with respect to the coordinates ${𝐱}_{i}$ of vertex *i*. It is often assumed that mechanical equilibrium is reached on a time scale much shorter than any driving process of tissue deformation [23]. Under this quasi-static assumption, the equation of motion simplifies to

$$0=\nabla_{i}\;E(𝐱).$$ (3)

The quasi-static Eq 3 can be solved by directly minimizing the energy function [23,39]. An alternative method, that has been used already in earlier works, is to evolve Eq 2 to steady state [22,48], in which case the friction term can be interpreted as a numerical relaxation method.

In the following, we describe the energy function that is commonly used in 2D apical vertex models to phenomenologically describe the intra- and inter-cellular mechanics of epithelial tissues [23]:

$$E(𝐱)=\underset{\text{area elasticity}}{\underbrace{\sum_{a}\frac{k_{A}}{2}{\left(A_{a}-A_{0}\right)}^{2}}}+\underset{\text{line tension}}{\underbrace{\sum_{\langle a,\;b\rangle }k_{l}\;l_{ab}}}+\underset{\text{perimeter contractility}}{\underbrace{\sum_{a}\frac{k_{P}}{2}P_{a}^{2}}}.$$ (4)

The three terms correspond to three types of forces typically considered to contribute to the sum of forces ${𝐅}_{i}$ acting on vertex *i* (Eq 1). The first term, summing over all cells *a*, penalizes the change of the current cell area *A*_*a*_ from a predefined target cell area *A*_0_, prefactored by an elasticity coefficient *k*_*A*_. This term, referred to as area elasticity, represents bulk forces resisting area or volume changes of the cell (blue arrows in Fig 1B). Examples include the hydrostatic pressure from the cytosol or osmotic pressure across the cell membrane. The second term is linear with respect to the cell boundary length and represents an effective surface energy between adjacent cells. This includes both cortical tension (linear part of the perimeter elasticity, red arrows in Fig 1B) and adhesion forces by the adherens junctions (yellow arrows in Fig 1B). It sums once over all cell-cell junctions, denoted by ⟨a,b⟩, an unordered pair of neighboring cells *a* and *b*. *k*_*l*_ is the tension coefficient, $l_{ab}:=\|{𝐱}_{i}-{𝐱}_{j}\|$ is the length of the contact surface between cells a, b , and vertices *i* and *j* are connected by edge ⟨a,b⟩. If cortical tension dominates adhesion, then *k*_*l*_ > 0, while if adhesion dominates, *k*_*l*_ < 0. The area elasticity and line tension terms were already proposed in an earlier vertex model by Nagai and Honda [22]. The extension of this energy function with a perimeter contractility term was first proposed in the seminal study by Farhadifar et al. [23] to better capture a characteristic feature of epithelial cells: the contractile actomyosin cytoskeleton that forms during embryonic development at the apical cortex of a cell and can be found as a belt-like structure in later stages of development and in mature tissues [23,43]. In the energy function, the perimeter contractility term represents a second-order effect of the cytoskeleton where *k*_*P*_ is the contractility coefficient and $P_{a}:=\sum_{b}l_{ab}$ is the perimeter of cell *a*.

Taking the partial derivative of the energy function with respect to a vertex position ${𝐱}_{i}$, we can write the total force on vertex *i* (Fig 1C) as:


$${𝐅}_{i}=-\sum_{a}\frac{\partial E}{\partial A_{a}}\frac{\partial A_{a}}{\partial {𝐱}_{i}}-\sum_{\langle a,\;b\rangle }\frac{\partial E}{\partial l_{ab}}\frac{\partial l_{ab}}{\partial {𝐱}_{i}}$$

=∑a∈𝒜iπa𝐧a,i+∑⟨a,b⟩a,b∈𝒜iτab𝐥ab,i, (5)

where ${𝒜}_{i}$ is the set of cells containing vertex *i* and ⟨a,b⟩ is again an unordered pair of neighboring cells. The expression is a decomposition of the force ${𝐅}_{i}$ into stresses $\pi_{a}$, $\tau_{ab}$ and strains ${𝐧}_{a,i}$, ${𝐥}_{ab,i}$. To simplify the expression, we define ${𝐥}_{ab,i}:=-\partial l_{ab}/\partial {𝐱}_{i}={\hat{𝐥}}_{ab,i}$ and ${𝐧}_{a,i}:=\partial A_{a}/\partial {𝐱}_{i}=(1/2)l_{a,i}{\hat{𝐧}}_{a,i}$. ${\hat{𝐥}}_{ab,i}$ is the unit vector along the edge between cells *a* and *b*, pointing away from vertex *i*, *l*_*a*,*i*_ is the length of the line segment connecting the two adjacent shoulder vertices to vertex *i* in cell *a* (dashed line in Fig 1C), and ${\hat{𝐧}}_{a,i}$ is a unit vector perpendicular to the line segment between the shoulder cells, pointing toward the outside of cell *a* (Fig 1C). This decomposition can be generalized and is instrumental in the statistical inference of cell and tissue stresses from experimental data [49–51]. Using Eq 4, the mechanical stresses can be interpreted as a cell pressure $\pi_{a}$ from area conservation and an edge tension $\tau_{ab}$ from cell-cell adhesion and cortical tension:

$$\pi_{a}:=-\partial E/\partial A_{a}=-k_{A}(A_{a}-A_{0}),$$ (6)

$$\tau_{ab}:=\partial E/\partial l_{ab}=k_{l}+k_{P}\left(P_{a}+P_{b}\right).$$ (7)

In the following, we introduce the second simulation step in the vertex model framework which aims to capture cell rearrangements in a confluent tissue. In the reference model, and many other models, these are implemented as update rules acting on the network’s topology [23,28,39]. Adopting terminology from foam physics, where similar phenomena exist [5], the first two transitions are called T1 and T2 which describe cell neighbor exchanges and cell extrusions, respectively. The third transition is specific to biology: a cell divides into two daughter cells. Here, we outline a set of specific procedures for performing the three common topological transitions in the vertex model (Fig 1D) [23,28,39]:

1. Cell neighbor exchange, also referred to as a T1 transition: If $l_{ab}<l_{\text{neighbor exchange}}$, where $l_{\text{neighbor exchange}}$ is a length threshold below which the bicellular junction is considered disintegrated, delete edge *ab* and merge attached vertices. Then, split the vertex again and create a new edge with a length $l_{cd}>l_{\text{neighbor exchange}}$ perpendicular to the old edge.
2. Cell extrusion, also referred to as a T2 transition: If $A_{a}<A_{\text{extrusion}}$, where $A_{\text{extrusion}}$ is a minimum cell area, merge all vertices that define cell *a* and eliminate cell *a*.
3. Cell division: If $A_{a}>A_{\text{division}}$, where $A_{\text{division}}$ is a critical cell area beyond which a cell divides, split the cell *a* into two new cells with a new edge, which intersects the original cell center at random orientation and creates two new vertices. The cell division rule is usually accompanied by a more or less simplistic cell cycle model, e.g., a time-dependent deterministic target cell area *A*_0_(*t*) that is increasing monotonically in time. In such a case, the decision threshold is set, e.g., to double the initial target area $A_{\text{division}}:=2A_{0}$.

It is evident that the transition rules involve sharp and artificial threshold values such as the minimum edge length for triggering a transition, and the length of the new edge after a T1 rearrangement. For now, we can interpret the thresholds as bound to precision limits in experimental observations. We note that the algorithms mentioned above are only exemplary for the three typical topological transition rules in vertex models and a variety of implementations exists [39,52]. We will discuss nuances of implementation choices in Sect 4.

Last, we highlight a physical perspective of the vertex model that has illuminated avenues to characterize and change material properties of a tissue [23,29,39,53,54]. One can rewrite the energy function in Eq 4 as

$$E(𝐱)=\underset{\text{area elasticity}}{\underbrace{\sum_{a}\frac{k_{A}}{2}{\left(A_{a}-A_{0}\right)}^{2}}}+\underset{\text{perimeter elasticity}}{\underbrace{\sum_{a}\frac{k_{P}}{2}{\left(P_{a}-P_{0}\right)}^{2}}},$$ (8)

by omitting a constant and defining a target cell perimeter $P_{0}:=-2k_{l}/k_{P}$ where the factor 2 accounts for the convention to only sum over each edge once [29,39,55]. In this alternative formulation, two important phase transitions are controlled by the target cell shape $p_{0}:=P_{0}/\sqrt{A_{0}}$ (Fig 1E): (1) Below $p_{0}^{hex}=2\sqrt{2}\sqrt[{4}]{3}\approx 3.72$, the ground state of the tissue, i.e., the global minimum of the energy function, is a lattice of regular hexagons. When *p*_0_ increases past $p_{0}^{hex}$, the regular hexagonal ground state becomes energetically unstable to deformation [23,39,56]. (2) In a disordered multicellular tissue, which is a metastable network in the vertex model, a solid-to-fluid transition occurs as *p*_0_ increases past a critical threshold $p_{0}^{*}$ [29]. This transition is characterized by the vanishing energy barriers for cell rearrangements, particularly T1 transitions, allowing the tissue to flow. As the two adjacent hexagons in a four-cell unit become pentagons while undergoing a T1 transition (Fig 1D), the shape index of the solid-to-fluid transition approximately corresponds to that of a regular pentagon $p_{0}^{*}\approx p_{0}^{pent}=2\sqrt{5}(5-2\sqrt{5})1/4\approx 3.81$ [29]. The exact value of $p_{0}^{*}$ can vary depending on the extent of cell packing disorder [10] and implementation details such as topological transition rules and energy minimization schemes [46,47].

Based on the reference model introduced here, we discuss extensions to this reference model in the next section.

## 3 Subcellular extensions of the vertex model

### 3.1 Spatiotemporal dependence of model parameters

**Fig 2:**
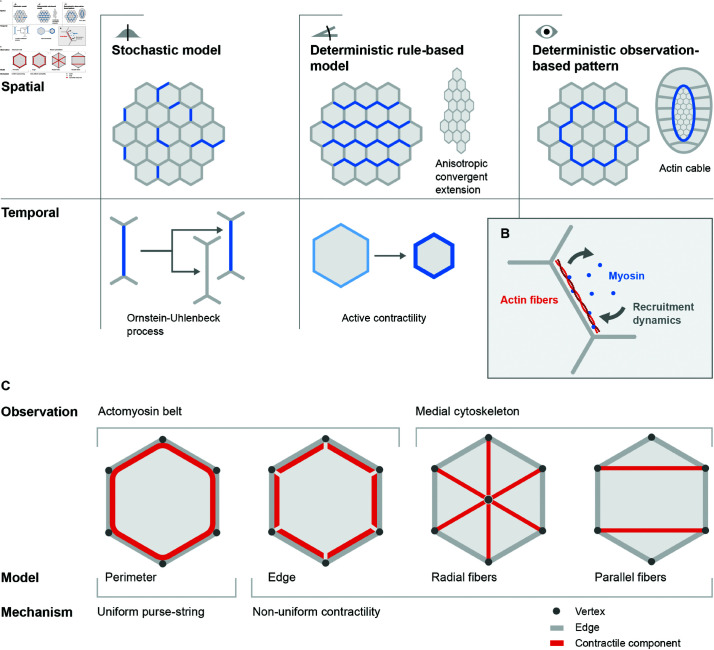
Spatiotemporal dependence of model parameters and alternative cell contractility models. (A) Different models with spatiotemporally dependent coefficients aim to represent observed effects of (B) the complex recruitment dynamics of myosin to actin fibers in the cytoskeleton. (A) Stochastic models can describe spatial static inhomogeneity of myosin distribution. This can be expanded towards temporal stochastic processes (e.g., Ornstein-Uhlenbeck) where myosin abundance on an actin fiber varies over time. Deterministic models impose a specific condition for myosin distribution, e.g., with respect to an angle or a clock. These models are directly motivated to capture observations like convergent extension or active contractility. Some observed patterns are implemented model-free into vertex model parameters. A representative example is the contractile cable along the perimeter of the amnioserosa during dorsal closure of *Drosophila* embryo development. (C) The actomyosin contractility mode expressed in a vertex model with a perimeter contractility term [23] is sometimes called a *purse-string* mechanism; it represents a continuous actomyosin belt along the cell perimeter and generates uniform contractility. Apart from that, a series of non-uniform contractility models exist. The edge model assumes a discontinuous actomyosin belt and thus reduces coupling of the contractility structures [80]. The medial cytoskeleton observed in premature tissues has been modeled as isotropic radial fibers and anisotropic parallel fibers [35,81,82]. Parallel fibers carry a load and resist isotropic extension without introducing additional degrees of freedom [82].

Protein activities in tissues are subject to thermal fluctuations and active regulations. In particular, the actomyosin cortex is subject to dynamic processes giving rise to contractile forces dependent on molecular turnover (Fig 2B) [57,58]. To incorporate temporal and spatial inhomogeneities in the mechanical properties of bicellular junctions, a straightforward generalization of the vertex model is to allow for a time-dependent, edge-specific line tension coefficient $k_{l}=k_{l,ab}(t)$ in the energy function with the perimeter contractility term (Eq 4) or the target perimeter $P_{0}=P_{0,a}(t)$ in the perimeter elasticity formulation (Eq 8) [44]. In the following, we categorize extensions that introduce parameter heterogeneity in the reference model by their spatial or spatiotemporal variation and group them according to their governing method: a stochastic model, a deterministic rule-based model, or a deterministic model-free observation-based pattern (Fig 2A). We summarize these extensions in Table 1, highlighting different model variants, their biological motivations, specifications of relevant parameters and conclusions from specific studies.

**Table 1:** Overview of vertex model extensions (Part 1).

Variant	Motivation	Parameter	Specification	Conclusion	References
**Spatiotemporal dependence of model parameters**					
**Stochastic spatial model**	Spatial variation of myosin activity	Tension coefficient	Gaussian or uniformly distributed	Uniform distribution of tensions does not change the cell packing [59].	[49,50,59]
	Influence of celleak heterogeneity on tumor invasion dynamics	Target perimeter	Gaussian distributed	Increased shear modulus and rigidity; heterogeneous solid state with distinct percolation properties.	[36,44]
Stochastic temporal model	Fluctuations ineak cellular parameters (e.g., myosin turnover with an unknown rate)	Tension coefficient	Governed by Ornstein-Uhlenbeckeak process	Higher amplitudes of edgeeak tension fluctuations fluidize tissues, while the fluctuationeak persistence time affects fluidization non-monotonically [37,64].	[7,37,60–65]
**Deterministiceak spatial condition for an observed pattern**	Supracellular actineak cables [54,70–75]	Tension coefficient	Predefined supracellular actin cable paths	Actin cable smoothens the rim of a tissue and contributes toeak compartmentalization. Cables are not a major driver of dorsal closure or salivary gland invagination.	[76–78]
**Rule-basedeak deterministic spatial model**	Planar cell polarityeak (PCP)	Tension coefficient	Orientation dependent (anterior-posterior)	Anterior-posterior oriented cell tensions bias T1 transitions in a specific direction, collapse edges into rosettes, and promote convergent extension.	[6,10,33,66]
	Planar cell chirality (PCC)	Tension coefficient	Orientation dependent (left-right)	Left-right asymmetric celleak tensions drive asymmetrical cell intercalation and organ rotation.	[67–69,83,84]
**Rule-basedeak deterministic temporal model**	Apical constriction during dorsal closure	Target perimeter	Time dependent (decreasing)	A decreasing target perimeter hinders tissue fluidization.	[36]
**Rule-basedeak deterministic temporal model with stochastic spatiotemporal activation**	Cell cycle	Target area	Time dependent (increasing)	Disorder introduced byeak cell division explains observed cell packing distributions.	[23]
	Active contractility from medial and peripheral actomyosin pools	Target area,eak target perimeter	Time dependent (decreasing)	The medial pool of actomyosin produces more anisotropic cells than an actomyosin ring.	[79]
**Alternative cell contractility models**					
**Edge-based**	Contractility varies anisotropically alongeak the cell perimeter.	Edge elasticityeak (replacement for perimeter elasticity)	Decoupling into independent edges	An anisotropic edge-based contractility model bettereak represents *Drosophila* pupal wing, notum, and embryo data than a perimeter-based model [41].	[32,34,35,41,45,81,83,84]
**Radial fibers**	An actomyosin meshwork in the apical cortex mediates contraction [43,85,86].	Additional contractile intracellular edges	Distributed radiallyeak connecting an additional central vertex with vertices on the perimeter	The medial pool of actomyosin could contribute to convergent extension and dorsal closure.	[32,35,45,81]
**Parallel fibers**	Number of stress fibers scales with cell area.	Additional contractile intracellular edges	Orientation dependent (aligned with major stress axis)	Fibers limit cell elongation under tension.	[82]

Note that some references contain multiple specifications. If no specific reference is provided for a motivation or conclusion, it is based on the respective list of references in the last column.

To capture spatial variation of myosin activity at different cell-cell junctions, *k*_*l*,*ab*_ can be drawn from a Gaussian distribution $k_{l,ab}∼𝒩(k_{l,0},\sigma_{l}^{2})$ with mean value *k*_*l*,0_ and variance $\sigma_{l}^{2}$ [49,50] or from a uniform distribution [59]. Independent of the specific choice of a distribution, stochastic target perimeters result in increased shear modulus and rigidity of tissues and lead to a heterogeneous solid state with distinct percolation properties which could be relevant for tumor invasion dynamics [44].

To capture temporal fluctuations in tension, for example due to protein binding and unbinding, *k*_*l*,*ab*_(*t*) can be described by an Ornstein-Uhlenbeck process

$$\frac{d}{dt}k_{l,ab}(t)=-\frac{k_{l,ab}(t)-k_{l,0}(t)}{\tau_{l}}+\xi_{ab}(t),$$ (9)

where $\xi_{ab}(t)$ is a Gaussian white noise with zero mean $\langle \xi_{ab}(t)\rangle =0$ and non-correlation between two edges *ab* and *cd*, $\langle \xi_{ab}(t_{1})\;\xi_{cd}(t_{2})\rangle =2\sigma_{l}^{2}/\tau_{l}\;\delta_{ac}\delta_{bd}\;\delta (t_{1}$ − *t*_2_) where $\tau_{l}$ is the relaxation time of the process [7,60–63]. Temporal fluctuations in tensile forces drive fluidization in tissues characterized by loss of correlation between tension and strain [37,64,65].

In other cases, spatial and temporal inhomogeneities are required to be deterministic. A classical example of deterministic spatial inhomogeneities is angle-dependent tension, which can be used to model planar cell polarity – the polarized distribution of protein activities within the plane of the tissue – as well as planar cell chirality, which involves the intrinsic asymmetry and directional movement of cells within the tissue. Anisotropic tensions can explain tissue convergent extension [10,33], the increase in the stability of four- and higher-order rosette vertices [6,66] and asymmetrical cell intercalation and organ rotation [67–69]. Spatial inhomogeneities are also often specified to a desired pattern. For example, linear bundles of actomyosin cytoskeleton, termed *cables*, span multiple cells. They play critical roles in morphogenesis, often contributing to closure and invagination events [54,70–75]. These supracellular cables can be modeled by imposing a higher tension coefficient along a series of consecutive edges [76–78].

Deterministic temporal programs have already been used in the model by Farhadifar et al. to include a cell cycle via increasing target cell areas before a cell division occurs [23], although the activation of a division cycle is initiated stochastically. Similarly, the decrease of target cell perimeters has been a successful model to capture the material properties of the amnioserosa of *Drosophila* during dorsal closure. Here, a combination of heterogeneous and decreasing target perimeters arrests fluidization of the tissue [36]. This suggests that stochasticity combined with deterministic growth or shrinkage is a common motif to explain observed tissue properties. A comparison between driven oscillations of target areas *A*_0_(*t*) and target perimeters *P*_0_(*t*) finds that area oscillations that represent the medial activity of actomyosin tend to produce more anisotropic cells, suggesting that different pools of contractility may produce distinct cell morphologies [79].

### 3.2 Alternative cell contractility models

The continuous actomyosin belt represented in the perimeter contractility model (Eq 4) produces uniform contractility along the cell boundary. In epithelial cells, however, the actomyosin displays distinct subcellular localizations that vary with confluence, functions, and developmental stages. Mature, confluent epithelial tissues predominantly exhibit a continuous, circumferential actomyosin structure along the cell perimeter, associated with adherens junctions [43]. In contrast, during early developmental stages, when confluency may be temporarily disrupted for large-scale tissue reorganization [87], actomyosin structures appear more medial, discontinuous, and heterogeneous—as has been observed in gastrulation of chick embryos [87] and *Drosophila* embryos [43,85]. Similar actomyosin structures have been observed in cell cultures of human intestinal [88] and Madin-Darby canine kidney (MDCK) cells [89]. These formations, often referred to as the *medial pool* of actomyosin, are essential for pulsed contractions and apical constriction [43,85]. To capture the aforementioned observations, some models incorporate non-uniform contractility (Fig 2C). Here, we consider two main types of these extensions, namely edge-based contractility and various medial contractile structures (summarized in Table 1).

Firstly, tricellular junctions are thought to pose a physical obstacle to the transport of proteins along the cell perimeter, and thus the contractile force may not be uniform along the cell perimeter [90–93]. Experimental studies indicate that each bicellular junction functions as an independent contractile unit, where actin cables are anchored end-on to cadherin complexes binding to tricellulin of the tricellular tight junctions ensuring tissue confluency [94,95]. Vertex models with edge-based contractility terms aim to individually describe bicellular junctions of a cell [32,34,35,55]. In an edge-based contractility model, the last term of the vertex model energy function is modified

$$E_{\text{edge-based}}(𝐱)=\underset{\text{area elasticity}}{\underbrace{\sum_{a}\frac{k_{A}}{2}{\left(A_{a}-A_{0}\right)}^{2}}}+\underset{\text{edge (line) tension}}{\underbrace{\sum_{\langle a,\;b\rangle }k_{l}\;l_{ab}}}+\underset{\text{edge contractility}}{\underbrace{\sum_{\langle a,\;b\rangle }\frac{k_{l}^{\prime }}{2}l_{ab}^{2}}}.$$ (10)

Here, in the third term, contractility is based on individual edges, with an elastic coefficient $k_{l}^{\prime }$. Due to the quadratic expression, this energy function is different from the perimeter contractility model (Eq 4), even when the line tension coefficient *k*_*l*_ and the contractility coefficient $k_{l}^{\prime }$ are constant for all edges. Analogous to the reformulation of the perimeter-based model (Eqs 4 and 8), one can absorb the line tension into the quadratic term in Eq 10 and introduce a target edge length *l*_0_. In doing so, the energy function takes a form, equivalent up to a constant:

$$E_{\text{edge-based}}(𝐱)=\underset{\text{area elasticity}}{\underbrace{\sum_{a}\frac{k_{A}}{2}{\left(A_{a}-A_{0}\right)}^{2}}}+\underset{\text{edge elasticity}}{\underbrace{\sum_{\langle a,\;b\rangle }\frac{k_{l}^{\prime }}{2}{\left(l_{ab}-l_{0}\right)}^{2}}}.$$ (11)

In 2D apical vertex models embedded in 3D space, an anisotropic edge-based contractility induces chiral cell sliding driving asymmetric twisting similar to what is observed in organs like the heart [83,84]. A vertex model with both perimeter contractility and edge contractility can capture mixed effects of contractile actomyosin at the cell border [80].

Further, some researchers incorporate additional contractile structures into the model to represent force-generating mechanisms that are not confined to cell perimeters, such as the star-like formations recently observed on the basal side of epithelial cells [96]. When studying the oscillations of cell areas in the process of dorsal closure during *Drosophila* development, a series of studies add radial stress fibers, termed *spokes*, connecting the vertices with the center of each cell [32,35,45,81]. The spokes are implemented as contractile, elastic intracellular edges. In addition to representing the medial pool of actomyosin, they also play a similar role to pressure forces due to area elasticity. These stress fibers are still isotropic in the sense that they are oriented along different directions well distributed in space provided that cell shapes are not strongly elongated. Some other studies explore the effects of anisotropic contractile structures. Parallel stress fibers aligned with imposed unidirectional stretching are employed in an effective force-based vertex model to explore the scaling of the number of fibers with cell apical areas [82]. Similarly, the dynamics of various subcellular stress fiber geometries in a stress-fiber reinforced vertex model reveal a size-dependent response of cells [97]. One comparative study confirms that edge-based contractility models belong to the same sub-class of vertex models as perimeter-based contractility models, exhibiting a crossover from soft to stiff network behaviors [42]. Other studies suggest that different contractility modes and structures might influence cell packing geometry and the threshold of a rigidity transition [79,82]. Another way to incorporate anisotropic contractility from the medial myosin pool is to write an anisotropic continuous stress in the bulk of the cells and derive their effective forces on the cell-cell junctions and vertices [99]. This type of model has been used to investigate how epithelial colonies elongate through collective effects, with tissue elongation resulting from anisotropy in the average cell elongation [99], and how the V-shaped muscle compartments emerge in zebrafish, revealing that spatially modulating the mechanical environment around and within tissues can lead to complex organ shape formation []. In these two examples, the anisotropic bulk stress is crucial for symmetry breaking during tissue growth and sculpting tissue shapes, respectively. However, recent comparative work has shown that the two different formulations that have been employed to derive the forces on vertices [99], lead to distinct tissue dynamics due to differences in their treatment of non-affine deformations [100]. Moreover, neither description of anisotropic bulk stresses could fully represent equivalent forces arising from cell-cell junctions in the reference vertex model [100]. Overall, these results suggest that different modeling choices could lead to distinct predictions, and it is important to align model design with experimental observations of contractile structures, which vary across biological contexts.

### 3.3 Subcellular regulation of cell contractility and mechanosensitive response

**Fig 3:**
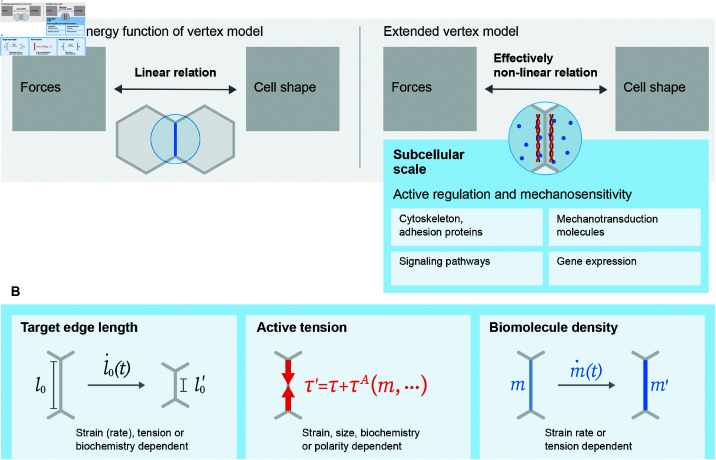
The effective relation between forces and cell shapes in the vertex model can be changed by introducing submodules featuring active regulation and mechanosensitive feedback describing activities at the subcellular scale. (A) The mechanics in the reference vertex model [23] are governed by an energy function (a phenomenological Hamiltonian) that dictates a linear relation between forces and cell shape (observed e.g., in the ground states or a proliferation-free simulation). To be able to capture realistic tissue morphologies, vertex models are commonly extended to a full simulation framework with components refining the subcellular scales, resulting in effectively nonlinear relations between forces and cell geometry [65]. The subcellular activities include the activation and turnover of cytoskeleton machinery and adhesion proteins, the relaying of mechanical signals through mechanotransduction molecules, the modulation of mechanical forces by the spatiotemporal dynamics of signaling pathways, and the activation and deactivation of genetic programs instructing protein synthesis. (B) Examples of submodules that actively regulate quantities are strain-dependent remodeling of the target edge length, edge length-dependent active tension, or strain-dependent remodeling of the density of a biomolecule, e.g., myosin. In systems with mechanosensitive feedback, these remodeled quantities are usually interdependent and formulated as coupled differential equations.

The reference model [23] describes cell-cell junctions as elastic springs driven to equilibrium by forces derived from an energy function Eq 4. In other words, the mechanical forces along the edges in the model are determined by the deformation of the polygonal tiling through a positive linear relationship. This simplified representation of the mechanical behavior of cell-cell junctions is hardly sufficient considering that the junctions are tightly regulated by complex, interacting subcellular elements such as cytoskeleton dynamics, mechanotransductive and other signaling pathways, and gene expressions. Two early seminal studies, using a lateral 2D vertex model, explore how deformation-dependent edge target length could lead to apical constriction and epithelial invagination [48,101]. The showcased mechanical self-organization suggests that tissues might leverage mechanical signals such as force or deformation to actively regulate their contractile forces. This type of *feedback loop* can result in spontaneous tissue configuration changes, a behavior loosely referred to as *mechanosensitivity*. With the advance in experimental and imaging techniques, knowledge of how cells actively control their mechanics on the subcellular scale provides increasingly solid foundations to the mechanosensitive hypothesis (Fig 3B) [11,82,91,102–110]. Together with the accumulation in experimental evidence, theoretical interest in mechanical self-organization has also grown rapidly [111].

The vertex model is a convenient tool to incorporate junction regulatory effects (Fig 3A). Cell division, as implemented in the reference model [23], is already an active event that reflects the cell cycle, an effect beyond the passive linear junctional tension dictated by the energy function, although it does not form an explicit feedback loop. One of the earlier studies combining vertex modeling and experiments on how mechanosensitivity contributes to morphogenesis encodes a feedback loop by letting the cell target parameters depend on the strain and suggests that strain-enhanced apical constriction is critical in the invagination of an optic-cup organoid [112]. Notably, the authors already deploy a 3D vertex model. This approach of implementing feedback loops in mechanics-dependent model parameters is a generalization of our first category of extensions with spatiotemporal dependence of model parameters. Meanwhile, it can be seen as a long-time limit of other implementations using differential equations to describe the evolution of model parameters in time, representing subcellular scale activities to study their effects at the tissue level. In more recent work, one approach, using the edge-based elasticity model (Eq 11), is to introduce remodeling of the target edge length *l*_0,*ab*_, described by the following generic formulation

$$\frac{d}{dt}l_{0,ab}=f\left(l_{0,ab},\;\tau_{ab},\;\epsilon_{ab},\;m_{ab},\cdots \right),$$ (12)

where *f* is a general edge regulation function. We use the subscript *ab* for all variables to emphasize that they can be edge-specific. On the left-hand side of the equation is the absolute rate of change of the rest length [113], or alternatively, can also be the relative rate of change [34]. On the right-hand side, a variety of regulation mechanisms can be incorporated by adopting different forms of *f*. For example, the actomyosin cytoskeleton is well-known to be viscoelastic [114]. Thus, $f=\tau_{ab}/\mu$ describes linear Maxwell viscoelasticity with viscosity μ. More importantly, the target edge length can be remodeled by the strain $\epsilon_{ab}=l_{ab}/l_{0,ab}\;-\;1$ or strain rate ${\dot{\epsilon }}_{ab}$ [65,113], which is an active regulation mechanism. This type of feedback is proposed to explain the mechanical ratcheting of cell-cell junctions [113]. A recent variant of this model shows that strain-regulated tension, together with mechanical memory dissipation implemented through the fluctuation of tension coefficients and the relaxation of tension and strain, leads to transiently stable higher-fold vertices (rosettes) [65]. Notably, in contrast to other mechanosensitive models, this model incorporates negative feedback, where tension decreases under edge extension and increases under edge contraction. The negative feedback is found to stabilize rosettes [65].

Biochemistry can be more explicitly described by considering the dependence of *f* on the quantity or activity of a certain molecule, generically written as *m*_*ab*_. In this case, an additional evolution equation is required to govern the dependent variable *m*_*ab*_, which can be written in the form

$$\frac{d}{dt}m_{ab}=g\left(\tau_{ab},\;\epsilon_{ab},\;m_{ab},\cdots \right).$$ (13)

In one example, *m*_*ab*_ denotes the average edge density of myosin motors, with Eq 12 describing the active walking of myosin motors contracting actin filaments under low tension and the slipping of motors under high tension, while the myosin density *m*_*ab*_, in turn, depends on the strain rate of the edge ${\dot{\epsilon }}_{ab}$ through Eq 13 [34]. In this case, tissue viscoelasticity and cell size variability emerge from the feedback between the strain rate of cell-cell junctions and myosin recruitment, coupled with junctional viscoelastic relaxation [34]. In a 3D vertex model, the effect of remodeling several mechanical parameters – the surface area elasticity, the target surface area, and the surface tension – on apicobasal polarity is investigated [115]. By combining a vertex model with the biochemistry of key proteins, the study shows that the mechano-polarity feedback loop can drive spontaneous tissue folding.

Besides remodeling the target edge length or the target cell perimeter, an additional or alternative scheme is to augment the edge tension with an active force. With the decomposition into cell pressure $\pi_{a}$ and edge tension $\tau_{ab}$ (Eq 5), one can extend the edge tension as

$$\tau_{ab}^{\prime }=\frac{\partial E}{\partial l_{ab}}+\tau_{ab}^{A}\left(m_{ab},\cdots \right)\;,$$ (14)

where the additional active force $\tau_{ab}^{A}$ is regulated by phenomenological laws describing subcellular processes. Similar to *f* in the edge target length remodeling formulation, $\tau_{ab}^{A}$ can depend on an array of factors. For example, an additional contractile force $\tau_{ab}^{A}>0$ can be added, which becomes active only when the cell area exceeds a threshold. This feedback enhances tissue strength and resistance to rupture [116]. Others assume that the active force is dependent on additional quantities, such as a myosin activity level (e.g., [35]). Persistent cell neighbor exchanges and convergent extension could arise from this type of mechanochemical feedback [32,45,117]. The active force term can also depend on a cell polarity vector. The coupling results in a chiral patterning when the tissue is confined on a spherical surface, demonstrating how mechanosensitivity interacts with topology [118]. In these cases, again new rate equations need to be introduced to govern the additional variables, either $\tau_{ab}^{A}$ itself or the biochemical species *m*_*ab*_. In general, both positive and negative feedback loops can be easily incorporated into the outlined framework.

Some researchers consider further complexities in the regulation of cell mechanics. For example, the coupling of a lateral inhibition model with a vertex model reveals how mechanics and biochemistry combine to instruct the alternating cellular pattern in the inner ear [119]. With further progress in imaging technology and discovering molecular processes underlying the transmission and interpretation of mechanical signals by biological systems, we believe that with these extensions (summarized in Table 2) we are only seeing the beginning of what might become a major part of vertex model studies.

**Table 2:** Overview of vertex model extensions (Part 2).

Variant	Motivation	Parameter	Specification	Conclusion	References
**Subcellular regulation of cell contractility and mechanosensitive response**					
**Model parameter depends on cell mechanical states**	Cells sense and modulateeak tissue formation across different scales.	Target cell perimeter	Strain dependent	Strain-triggered apicaleak constriction is critical for invagination in the morphogenesis of optic-cup organoids.	[112]
**Rate equationeak governs the evolution of cell-cell junctions**	Cell-cell junctions remodel due to mechanical signals.	Target edge length	Strain dependent	Mechanical feedback can lead to ratcheting of cell-cell junctions.	[113]
			Strain rate dependent	Recapitulates stable rosettes like observed in tissues.	[65]
			Dependent on myosin density and aeak biochemical circuit	Reduction of target edge length promotes cell intercalation.	[45]
			Tension and myosin density dependent	Tissue with viscoelasticeak properties and increased variability of cell size.	[34]
	Epithelial folding shifts cell polarity which instructs mechanical forces.	Surface elasticity,eak target surface area,eak surface tension	Cell polarity dependent	Mechanopolarity feedback can lead to spontaneous tissue folding.	[115]
**Augmented force**	Myosin recruitment creates a contraction force.	Additional active force in edge tension term	Dependent on myosin density or a biochemical circuit or both	Dynamics of biochemicaleak proteins can lead to persistent cell neighbor exchanges andeak convergent extension, and can explain the oscillations and ratcheting of cells in dorsal closure.	[32,35,45,81]
			Strain dependent	Higher frequency of T1 events and wider distribution of polygon classes.	[65]
			Dependent on cell area	Propagating contraction pulses and resistance to rupture.	[116]
	Cells move collectively on a substrate.	Additional traction force term in equation of motion	Cell polarity dependent	Emergence of chirality when tissue is restricted on a spherical surface.	[118]

Note that some references contain multiple specifications.

## 4 Properties of extended vertex models

**Fig 4:**
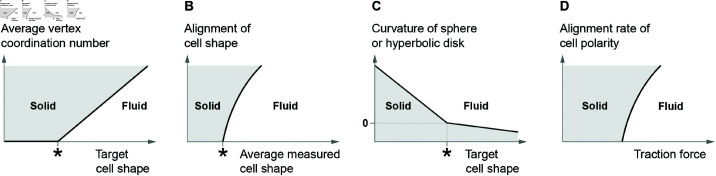
Dynamical phase diagrams describing a solid to fluid transition in various extended vertex models. (A) Average vertex coordination number (from $\bar{z}=3$ to $\bar{z}=6$) against target cell shape *p*_0_ in a model with active T1 transitions [120]; (B) alignment of cell shape against average measured cell shape $\bar{p}$ in an anisotropic tension model [10]; (C) Gaussian curvature against target cell shape in a vertex model restricted to a sphere (positive curvature) or hyperbolic disk (negative curvature) [121,122]; (D) alignment rate of cell polarity against traction forces in a traction force model on a spherical surface [118]. Asterisks indicate the critical target cell shape $p_{0}^{*}=3.81$ or average measured cell shape $\bar{p}=p_{0}^{*}$) in the reference model [29,39].

With emerging model variations, an important question is how different models preserve certain physical properties compared to the reference model [23]. In this section, we discuss extended vertex models, focusing on tissue properties, including extensions that alter these properties through means other than subcellular dynamics, structures, and feedback. One property of significance is tissue rigidity. As reviewed in Sect 2, it is well known that the reference model displays a rigidity transition between fluid and solid characteristics with changing line tension and perimeter contractility (*k*_*l*_ and *k*_*P*_ in Eq 4, respectively) [23,29]. This transition has been found to occur at a critical target cell shape $p_{0}^{*}$ (Fig 1E) [29,53]. Imaging data from tissues without directed stresses reveal that the cell rearrangement rate undergoes a sharp transition at a critical average cell shape $\bar{p}$. This threshold corresponds to the critical target cell shape in the vertex model [10]. In tissues with a source of anisotropy, however, the cell shape alone does not suffice to describe or control rigidity. Under anisotropic conditions it is possible to obtain ordered, rigid states with elongated hexagonal cells beyond the threshold where a regular hexagonal lattice in an isotropic tissue loses order and rigidity. As a result, rigidity must be evaluated taking into account other quantities, e.g., cell shape alignment [10]. Several other observables and control parameters have a profound impact on the solid-to-fluid phase transition previously characterized by the target cell shape alone, resulting in higher-order transitions (Fig 4A,4B and 4D) [10,118,120]. Additionally, 2D vertex sheets restricted on spherical surfaces, as is the case in many realistic biological scenarios such as embryos and organoids, lead to tissue fluidity becoming a function of Gaussian curvature (Fig 4C) [46,121,122]. A similar effect is seen in a vertex model where edges take the shape of circular arcs based on the Young-Laplace law [123]. Here, the solid-to-fluid transition already occurs at a less elongated target cell shape of *p*_0_ = 3.73. A recent review highlights different physical mechanisms that cause the collective arrest of cell motion in tissues [30]. While vertex models have been primarily devoted to studying tension-driven rigidity so far, “freezing” a tissue by reducing parameter fluctuations and cell motility is another possible mechanism of rigidification in systems featuring a source of activity, e.g., a cell cycle or traction forces [63,118]. Disentangling contributions to rigidity is not trivial, especially in active vertex models where both mechanisms are at play [30].

The reference vertex model [23] and extended vertex models are used to explore cell packing geometry and reveal mechanisms for the characteristic stability of *rosettes* – vertices which belong to five or more cells [28]. Packings and rosettes are observable in experiments and are closely related to tissue rigidity [29,120]. For cell packing geometry in general, one way to generate disorder in the vertex model is to enable programmed growth and division on randomly selected cells [23]. Recent studies demonstrate that tissues with realistic cell packing can emerge from models without stochastic cell proliferation [36,65]. Instead, these models incorporate stochastic tension coefficients along with either cell shrinkage or mechanosensitive feedback between edge tension and target edge length. This indicates that gradual deterministic changes with a source of stochasticity appear to be a common motif in causing tissue disorder. On the other hand, there is evidence that different contractility models can lead to distinct cell morphologies [79]. At the same time, cell packing disorder is closely related to tissue rigidity by modifying its transition threshold [10,44]. Therefore, it remains to be explored how specific model extensions that have emerged recently influence cell packing and their implications for tissue rigidity. Among various cell packing features, four-fold vertices and higher-fold rosette vertices are of particular interest. The average vertex coordination number $\bar{z}=\sum_{i}^{V}z_{i}/V$, summed over all *V* vertices, measures the density of rosettes, and in general cell packing disorder. The presence of higher-fold vertices, similar to isotropic cell shapes, is a good indicator for a rigid tissue [10,120]. A hexagonal lattice has $\bar{z}=3$, and the higher $\bar{z}$ is, the higher the critical target cell shape beyond which the tissue becomes fluid (Fig 4A) [120]. Rosettes can be identified from experimental images relatively easily, thus providing another observable for tissue rigidity. In regular T1 transitions in the vertex model, the four-fold vertex state is energetically unstable and thus short-lived (Sect 2 and Fig 1D). In epithelial tissues, however, four-fold vertices and rosettes are frequently observed. The unexpected stability of higher-fold vertices is a subject of study where multiple of the reviewed extensions have explanation power. High rosette stability can be achieved by low amplitude, high-frequency fluctuations of edge tensions such that edges are virtually trapped inside a T1 transition [37,64]. Similarly, delayed T1 transitions, a consequence of the finite time scales required by molecular complexes to undergo rearrangements, increase the frequency and stability of rosettes and make tissues more elastic [66]. Increased stability of rosettes can also be linked to tension fluctuations arising from mechanosensitive feedback between tension and target edge length, independent of implementation details of T1 transitions [65]. These studies offer insights into how the frequency and stability of rosettes synergize with model extensions including fluctuations and T1 delay time. They constitute the beginning steps for investigating the relationship between higher-fold vertices and more sophisticated extensions such as non-uniform contractility and mechanosensitive models.

The developments centered around tissue rheology and geometry imply that extensions of the vertex model can profoundly change predicted tissue properties, highlighting the need for comparative studies and unified views. Here, we identify three major lines of research. The first one continues along the subject of rigidity. A major result is the classification of the rigidity transition in the reference model [23,29] as one particular case of a broad class of rigidity arising from geometric incompatibilities that can be described by a minimal length [47]. Whether different vertex model variants display a rigidity transition has only been investigated recently by comparison of shear moduli in the quasi-static limit of vanished shear [42]. The authors have concluded that (i) the confluent foam model without the quadratic contractility term (see Sect 2) is unstable in the regime where the vertex model is in a fluid state; (ii) vertex models with edge contractility (Eq 10) [32,34,35,45,81] behave similarly to perimeter-based models (Eq 4) for small values of the area compressibility *k*_*A*_ in displaying a transition from fluid-like to solid-like when *l*_0_ is decreased. They have also suggested the uncertainty of a quasi-static limit for a model with thresholded yielding and tension remodeling [113], which likely requires a stationary state that is constructed differently. A recent study generalizes the theory of elastic properties of athermal, under-constrained systems [47] to include thermal fluctuations in an edge-based contractility model without area elasticity (*k*_*A*_ = 0). In thermal, under-constrained systems, it shows that energetic rigidity (also present in athermal systems) and entropic rigidity (the limit of infinitely stiff springs) “add up” like being connected in series [124]. This novel view unifies the physics of vertex models with that of polymer networks.

The second line, which we believe holds potential for further exploration, is the influence of topological transition rules and more generally the effects of sources of activity. The effects of sharp thresholds and their potential effects and artifacts on tissue dynamics have been addressed in a small number of studies [39,52,66]. Different algorithms of topological transition have been compared in [39]. Here, a simple T1 transition algorithm (detailed in Sect 2) is compared with more complex T1 procedures, where transitions are selected based on the most energetically favorable changes in the network. While similar in polygon distributions and cell area variations, energy-based algorithms are less numerically stable because they depend on very fine changes in energy landscapes. Additionally, the study reveals that omitting quasistatic cell growth prior to applying a cell division rule (outlined in Sect 2) does not affect results significantly. A study comparing a large range of implementation choices finds that simulation outcomes are robust to variations in T1 edge lengths, T2 transition thresholds, and simulation step order [52]. However, the study also reveals that the cell cycle duration and the interplay of growth, division, and rearrangements influence the tissue size and mechanics. Further, the time step size influences rearrangement dynamics and higher-order methods do not improve convergence. As topological transitions affect rosette stability and tissue rigidity [37,64,120], simulation timing choices can significantly impact tissue behavior [52,66]. From a broader perspective, topological update rules belong to a class of elements that drive tissues out of thermal equilibrium, thereby making them *active*. Examples of such elements include a growth and division rule, biochemical circuits, and mechanical feedback loops. In the long term, it will be instructive to categorize active elements and clarify their effects on model behavior. So far, some authors have emphasized “active” components, e.g., an active vertex model with cell self-propulsion [125] or actively oriented cell divisions [126], while others have not stated so explicitly even though their models include similar elements such as cell division which in itself can be considered an active process [23]. A clear and unified terminology distinguishing active and passive elements could pave the way toward a better understanding of the potential effects of different model components.

The third line, where further research can bring a significant understanding of tissue dynamics, is how different time scales interact. Many studies mentioned above already explore temporal effects [64,66,79]. In one recent comparative study, different friction coefficients used in the vertex model lead to distinct results in cell growth [127]. This is because the time scale of viscous relaxation arising from the friction with the ECM polymer substrate competes with intrinsic cellular time scales such as the cell cycle length and duration of growth. For models incorporating more complicated dissipation mechanisms such as tensor friction coefficients [128–130], there could be multiple viscous time scales to consider. Other models include internal dissipation from, e.g., area, edge and perimeter dissipation [128], neighbor-neighbor dissipation [130], and vertex-center viscosity [131]. However, as highlighted in an earlier review [27], dissipation processes in the vertex model and tissue mechanics remain poorly understood, and for vertex models employing over-damped motion as a numerical tool to solve energy minimization problems, caution is needed to ensure artificial viscous time scales do not distort results [27,52,127]. For models without a viscous time, other time scales, particularly those introduced by mechanosensitive feedback (Eqs 12 and 13), may be relevant. Future investigations explicitly addressing the interaction of such time scales could provide key insights into tissue dynamics.

Examining the properties of tissues raises the question of whether a unified theoretical framework for the vertex model can be constructed – one that could encompass simplified versions under different conditions and clarify how these simplified versions correspond to existing model parameters. Could a universal theory for vertex models emerge, akin to the ideal gas law in thermodynamics or the Navier-Stokes equations for fluid dynamics? The phenomenological Hamiltonian provides a promising foundation and has already yielded significant insights, particularly in understanding tissue rigidity. However, systematic, comparative studies remain limited, though they are essential to furthering both the practical applications of vertex models and our understanding of tissue mechanics.

## 5 Inferring mechanical stresses and properties in cells and tissues

Inference methods using statistical models to estimate mechanical stress in tissues are an orthogonal approach to mechanistic vertex models. The vertex model and other models discussed later in this review, e.g., the Voronoi model, can be classified as mechanistic models. Mechanistic models are designed to capture hypothesized mechanisms that are able to explain experimental observations. Statistical methods, on the other hand, aim for a statistical model that is able to capture the structure of observational data. In a confluent network of cells, this translates to a statistical method that, based on cell geometries extracted from cell boundary segmentations from experimental data, infers effective cell boundary tensions $\tau_{ab}$ and cell pressures $\pi_{a}$ (Eq 5). This method assumes local force balance in the bicellular junction network, similar to the vertex model (Eq 1) [49,50,132–134]. This makes it possible to compare predictions by vertex models based on specific phenomenological energy functions (e.g., Eq 4B) to estimates of effective forces (Fig 1C). A recent study includes inference methods into their model design process by first estimating how stresses in a tissue relate to strains [41]. By an information criterion, they find that in *Drosophila* pupal wing, pupal notum, and embryo an anisotropic edge-based contractility model (Eq 10) describes the relation between forces and cell shape better than the conventional perimeter-based model (Eq 4). They find that positive feedback between junction tension and shrinkage increases at specific developmental stages. Future studies will profit from including inference methods into their model design process by first estimating stresses in a tissue to characterize nonlinearities in the relation between stress and strain (Fig 3). Moreover, because stress inference is non-invasive, it can be used to estimate local stresses from a time-lapse dataset [49,132]. Mapping stress estimates in space and time enables comparison to vertex model simulations based on effective forces on a vertex, namely the changing forces that a specific tricellular junction is exposed to over a course of minutes or hours. In general, estimates of stresses during topological transitions could be helpful to profile energy barriers that are otherwise intractable with commonly used destructive laser ablation measurements. Details of stress inference methods and applications have been discussed in a previous review [51].

Furthermore, a novel approach aims to infer flow properties of tissues from their geometry and dynamics, based on vertex model simulations [40]. The method relies on the ratio of the strain rate increment and the stress increment to distinguish between regimes. Comparing results from conventional vertex model simulations in the solid phase with *Drosophila* pupal wing data, the study finds that the distribution of bond lengths can serve as a proxy for local distances to yield stress and thus reveal the regime in which flow is occurring [40]. This represents a promising direction of research on inference of tissue properties by leveraging a deeper understanding of the vertex model.

## 6 Complementary models for epithelial tissues

Here, we provide a short overview of several other types of models that are related to and complement the vertex model by providing alternative approaches to tissue mechanics at different spatial resolutions, degrees of freedom, and levels of details in the mechanics. Comparison with related models provides valuable insights into the strengths and limitations of the vertex model in resolving large system sizes, adhesion dynamics, and cell shapes.

### 6.1 Continuum models capturing vertex model properties

At macroscopic scales, continuum models, coarse-grained from vertex models, provide a possible means to bridge cellular-level mechanics with large-scale tissue behavior, without resolution of single cells. They formulate partial differential equations governing the evolution of several field functions, e.g., displacements, stress, strain, and strain rate, and are thus amenable to a variety of analytical and computational tools. In this way, tissues at macroscopic scales consisting of far more cells than in a vertex model can be studied. There have been many attempts at deriving the continuum limits of various elements of the vertex model. An energy function was formulated in terms of the strain tensor, and a solid-solid transition due to geometric frustration has been uncovered [135]. A normal mode analysis can be applied to the vertex model to compute the linear loss and storage moduli, revealing complex linear viscoelastic rheology of a 2D tissue [129,136]. Full dynamical theories could be derived by directly postulating the equations of elasticity or hydrodynamics and fitting at least part of the parameters to vertex model simulations or experiments [61,137,138]. They can also be constructed by homogenization techniques [77] or thermodynamic formalisms including Onsager’s approach [139], Poisson brackets [140] and a kinetic theory formulating a Fokker-Planck equation [141]. Such procedures can often give relations between macroscopic material parameters and vertex model parameters. A detailed survey of these studies and their predictions compared to each other and those from the vertex model are beyond the scope of this review. We point out, however, that these models focus on coarse-graining the reference model [23], and little is known about the continuum limits of extended vertex models like the ones discussed in Sect 3.

### 6.2 Vertex-like models with different resolutions of cell-cell junctions

**Fig 5:**
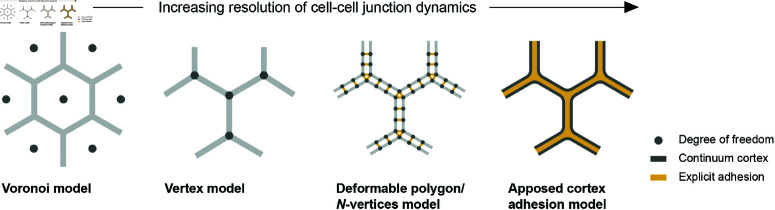
Vertex-like models by resolution of cell-cell junction dynamics. Increasing resolution of cell-cell junctions from left to right: The Voronoi model is a coarse-grained version of the vertex model with cell centers as degrees of freedom and implicit treatment of T1 transitions [125,142,143]; the vertex model as reference [28]; the deformable polygon or *N*-vertices model resolves the cell cortex by expanding the definition of vertices to a subjunctional flexible chain with explicit adhesion between cell membranes [7,13,148–151]; the apposed cortex adhesion model focuses on adhesion dynamics of cell-cell junctions during topological transitions with viscoelastic continuum cortices adhering to each other by an explicit continuous adhesion energy [152].

Several models can be considered “vertex-like” models in that they use a vertex model energy or strongly focus on resolving cell-cell junctions and topological transitions (Fig 5). The Voronoi model is a coarse-grained version of the vertex model with cell centers as degrees of freedom. It relies on the duality between the Delaunay triangulation of cell centers and the Voronoi tesselation, which resembles a membrane network, to update the equations of motion of the cell centers based on a vertex model energy function (Eq 8). It offers lower implementation costs due to the implicit treatment of topological transitions at similar computational cost (𝒪(NlogN) with *N* degrees of freedom) [125]. However, in the 2D case, the Voronoi model lacks a solid-to-fluid transition due to constraints imposed on cell areas and perimeters [125,142–144]. Similarly, a comparative study between 3D vertex models and 3D Voronoi models suggests broader distributions of cell shapes in vertex models, highlighting the power of vertex models in capturing heterogeneity in tissue properties [144]. Although this restricts the benefit of Voronoi models to mostly solid 3D systems, in fact many systems of interest in biology belong to this category [27,118,145–147]. At finer scales, models like the *N*-vertices models [7,13,148–150], the deformable polygon model (DPM) [151], and the apposed cortex adhesion model (ACAM) [152] allow for curvature, slippage and delamination of cortices, offering a more realistic description of cell-cell contacts. Recent experimental findings support the notion of a compression-dominated solid, which is observed in DPM simulations as a tension-to-compression transition between two solid regimes [151,153]. In general, further investigation is needed to distinguish between different phase transitions, described by various terms, such as epithelial-to-mesenchymal transition (EMT), jamming, rigidity, or percolation transition. Many of the observed phase transitions in epithelial tissues are not exclusively driven by only one of the commonly discussed mechanisms: cortical tension, cell-cell adhesion, tissue density or cell motility. Disentangling multiple mechanisms is of particular interest in, e.g., tissues undergoing embryonic development or cancer progression [30,153–155]. Multi-phase field models and continuum approximations of cell membranes provide avenues for exploring topological transitions, energy landscapes and continuous cell shape changes in epithelial tissues [156–160]. Moving forward, comparing and coupling model extensions and variants at different length scales and benchmarking them against material property studies in other models, such as the cellular Potts model, can offer deeper insights into tissue mechanics and behavior [161–164].

## 7 Software for vertex and vertex-like models

As vertex models become increasingly complex and more popular with practitioners across disciplines – specifically mathematics, physics, engineering, biology, and health sciences – the development of accurate, extensible, scalable, and accessible computational libraries becomes crucial. A variety of software packages caters to different needs in a diverse community of modelers (summarized in Table 3). Surface Evolver is a standard solver that computes the geometry of any given surface by minimizing an associated energy function using gradient descent, accommodating arbitrary topology, volume constraints, boundary constraints, and boundary contact angles [165]. It continues to be used to solve the quasi-static equations of motion, especially for models based on the reference model (eqs 2 and 8) [29,39]. Cell-based Chaste is a submodule of the Cancer Heart and Tissue Environment, designed for comprehensive cell population modeling [166,167]. It integrates cell cycle, cell motion, and biomolecule transport models, supporting various frameworks, including lattice-free models (vertex, Voronoi, overlapping spheres, and immersed boundary models), and on-lattice models (cellular automata and cellular Potts models). SAMoS is a Delaunay-based Voronoi model leveraging Delaunay-Voronoi duality for efficiency, as it avoids recalculating the entire Voronoi tessellation at each time step [125]. cellGPU implements a GPU-accelerated vertex model that is approximately ten times faster than its CPU counterpart and a hybrid GPU/CPU Voronoi model offering a speed increase of up to three orders of magnitudes for tissues in the topological transition-free solid regime [168]. TiFoSi is a simulation tool for planar epithelial cellular dynamics, integrating mechanosensitive feedback, gene expression, and interactions between cell populations. It allows customizable cell cycles, cleavage properties, protein partitioning during division, and cell communication [169]. Tyssue is a Python package for 2D and 3D vertex models, offering a flexible API for rapid model development and a range of energy function terms [170]. It also supports 2D apical surfaces embedded in 3D as well as full 3D modeling. The Vertex Model Solver of Tissue Forge enables event-based modeling and integrates vertex models with particle-based and subcellular methods, offering real-time simulation visualization [171]. neoVM is an implementation of a graph vertex model that utilizes knowledge graphs and a non-redundant database to streamline topological transitions [172]. It executes graph transformations through predefined queries, simplifying implementation and data handling. Most of the available software packages are implemented under an object-oriented paradigm and provide generalized representations of energy functions and numerical solvers in a modular fashion that make it convenient to couple to stochastic models and ODEs that govern a vertex model parameter. The diversity of these libraries manifests the popularity of vertex models in studies of tissue mechanics. We expect their capabilities to be continuously enhanced and expanded. Comparative studies of vertex models and their fundamental properties, as envisioned in earlier sections, could contribute to more comprehensive and generalized software packages.

**Table 3:** Overview of software packages for vertex and vertex-like models.

Software package	Programming language	Features	References
Surface Evolver	C (executable programs available)	Evolves surfaces toward minimal energy via gradient descent;eak handles arbitrary topology; supports volume and boundary constraints;eak accommodates boundary contact angles.	[165]
Cell-based Chaste	C++ (Python wrapper via pyChaste)	Models cell cycle, cell types, and signaling molecules; supports lattice-free models (vertex, Voronoi, overlapping spheres, immersed boundary) and on-lattice models (cellular automata, cellular Potts).	[166,167]
SAMoS	Python and C++	Delaunay-based Voronoi model using Delaunay-Voronoi duality; efficient by avoiding recomputation of the full Voronoi tessellation at each step.	[125]
CellGPU	C++/CUDA	Fully GPU-accelerated vertex model: ∼10× faster than CPU; hybrid GPU/CPU Voronoi model: up to $10^{3}\times$ faster in the solid regime.	[168]
TiFoSi	C++ (Python parser for XML configuration files)	Mechanosensitive feedback; customizable cell cycle and cleavage properties; protein number partitioning during cell division.	[169]
Tyssue	Python	Supports 2D apical vertex models embedded in 3D and full 3D models; provides an API for modeling.	[170]
Tissue Forgeeak vertex model solver	C++ (Python API available)	Event-based modeling; integrates vertex models with particle-based and subcellular methods; real-time simulation visualization.	[171]
NeoVM	Python	Non-redundant graphs for topological transitions; graph transformations via predefined queries.	[172]

## 8 Conclusion

In this review, we have surveyed recent advancements in the vertex model. We find that extensions to a seminal vertex model [23] have largely aimed at coupling tissue mechanics with model constructs describing subcellular activities. These extensions are made possible through several types of implementations: local and temporal dependence of model para-meters, stochastic fluctuations, non-uniform or non-junctional contractility structures, and coupled biochemical and mechanical regulatory mechanisms. Vertex models have been successfully applied to obtain insight in both tissue properties in general and the mechanics of biological processes, specifically tissue dynamics, e.g., dorsal closure [35,36,76,78], convergent extension [6,10,32,33,35,45,66], and tumor invasion [36,44].

In synthesizing the predictions on tissue properties, we point out that different models could lead to similar results in certain aspects, such as the generation of disorder by either growth or shrinkage coupled to stochasticity [36,65]. In other aspects, however, distinct extensions can lead to significant differences in properties, such as cell morphologies and tissue rigidity transitions. This leads to questions regarding their comparability and the quest for a unified theoretical framework, which have just started to be addressed [6,41,42,47]. We also call for attention to several model components whose effects have not been sufficiently examined, including different time scales in the model and topological transitions as a source of activity.

Statistical inference methods, based on a cellular network geometry and a force balance assumption, provide an orthogonal approach to estimating mechanical parameters from experimentally measured cell geometries, enabling data-driven model selection, force estimates, and model comparisons in epithelial tissues [41,49,51,134]. Furthermore, we present a comparison of several other models to the vertex model. Continuum models, coarse-grained from vertex models, enable the study of large-scale tissue behavior by bridging cellular mechanics and macroscopic properties using analytical and computational tools, but their applicability to extended vertex models remains largely unexplored. Models similar to the vertex model in resolving cell shapes include the Voronoi model, the *N*-vertices model, the deformable particle model, and the apposed cortex adhesion model [144,151,152]. These models differ from vertex models in how they resolve cell-cell junction dynamics, which affects their tissue-scale behavior and emphasizes the multiscale nature of tissue dynamics. As vertex models gain popularity, the development of accurate, extensible, scalable, and accessible computational libraries like Surface Evolver, Cell-based Chaste, SAMoS, CellGPU, TiFoSi, Tyssue, Tissue Forge, and NeoVM has become crucial to facilitate simulations of biological processes across disciplines.

With further collaborative effort across experimental, computational, and theoretical communities, we expect the vertex model to become a well-formalized and robust framework to study how the physical properties of tissues are influenced by kinetics and dynamics at the subcellular scale. It is evident that in recent years, there has been significant progress in extending vertex models and studying their properties. We expect that research in vertex models will continue advancing in multiple directions. First, the diversity of extensions will continue growing to incorporate additional mechanical and biochemical processes now accessible by experimental approaches such as optogenetics [15]. Of particular interest could be adapting vertex models to further study different developmental stages [43] and more complex cell topologies which require sophisticated 3D models [145,147,172]. Second, comparative studies are essential to clarify the differences in vertex models equipped with different extensions. Through these studies, it could become clear which physical properties of tissues are conserved to form a well-defined class of vertex models and how properties are potentially lost [42]. One established aspect is tissue rigidity. On the other hand, topological transition rules and their effects remain underexplored and could benefit from systematic works similar to [6,65,66]. Third, the development of a multiscale approach in many recent vertex models presents opportunities to unravel the dynamics of epithelial tissues comprehensively and bridge molecular processes to the scale of organs. In particular, integrating submodules at smaller scales effectively supplies parameters at the cellular scale, e.g., tension coefficients [34,35]. Four, more extensive comparisons between model predictions and experimental data would enhance model predictability and fidelity, allowing for deeper insights into tissue dynamics, and potentially illuminating how tissue properties could be engineered for medical applications. We believe the vertex model framework holds promise for advancing our understanding of epithelial tissue mechanisms, morphodynamics, and properties by integrating subcellular structure, dynamics, and feedback.

## References

[1] Bryant DM, Mostov KE. From cells to organs: building polarized tissue. Nat Rev Molecul Cell Biol. 2008;9(11):887–901. doi: 10.1038/nrm2523 PMC2921794 18946477

[2] Mateos C, Valencia-Expósito A, Palacios IM, Martín-Bermudo MD. Integrins regulate epithelial cell shape by controlling the architecture and mechanical properties of basal actomyosin networks. PLOS Genet. 2020;16:e1008717. doi: 10.1371/journal.pgen.1008717 32479493 PMC7263567

[3] Kim EJY, Sorokin L, Hiiragi T. ECM-integrin signalling instructs cellular position sensing to pattern the early mouse embryo. Development. 2022;149(1):200140. doi: 10.1242/dev.200140 34908109 PMC8881741

[4] Li ZS, Adams JG, Chisholm AD. Form and function of the apical extracellular matrix: new insights from Caenorhabditis elegans, Drosophila melanogaster, and the vertebrate inner ear. Faculty Rev. 2020;9. doi: 10.12703/r/9-27 PMC7886070 33659959

[5] Weaire D, Hutzler S. The physics of foams. Oxford: Oxford University Press; 2000

[6] Spencer MA, Jabeen Z, Lubensky DK. Vertex stability and topological transitions in vertex models of foams and epithelia. Eur Phys J E. 2017;40(1):2. doi: 10.1140/epje/i2017-11489-4 28083791

[7] Kim S, Pochitaloff M, Stooke-Vaughan GA, Campàs O. Embryonic tissues as active foams. Nat Phys. 2021;17:859–66. doi: 10.1038/s41567-021-01215-1 34367313 PMC8336761

[8] Campàs O, Noordstra I, Yap AS. Adherens junctions as molecular regulators of emergent tissue mechanics. Nat Rev Mol Cell Biol. 2023;25(4):252–69. doi: 10.1038/s41580-023-00688-7 38093099

[9] Messal HA, Alt S, Ferreira RMM, Gribben C, Wang VM-Y, Cotoi CG, et al. Tissue curvature and apicobasal mechanical tension imbalance instruct cancer morphogenesis. Nature. 2019;566(7742):126–30. doi: 10.1038/s41586-019-0891-2 30700911 PMC7025886

[10] Wang X, Merkel M, Sutter LB, Erdemci-Tandogan G, Manning ML, Kasza KE. Anisotropy links cell shapes to tissue flow during convergent extension. Proc Natl Acad Sci U S A. 2020;117(24):13541–51. doi: 10.1073/pnas.1916418117 32467168 PMC7306759

[11] Kaliman S, Hubert M, Wollnik C, Nuić L, Vurnek D, Gehrer S, et al. Mechanical regulation of epithelial tissue homeostasis. Phys Rev X. 2021;11(3):031029. doi: 10.1103/PhysRevX.11.031029

[12] Roshal DS, Martin M, Fedorenko K, Golushko I, Molle V, Baghdiguian S, et al. Random nature of epithelial cancer cell monolayers. J R Soc Interface. 2022;19(190):20220026. doi: 10.1098/rsif.2022.0026 35537474 PMC9090488

[13] Weng S, Huebner RJ, Wallingford JB. Convergent extension requires adhesion-dependent biomechanical integration of cell crawling and junction contraction. Cell Rep. 2022;39(4):110666. doi: 10.1016/j.celrep.2022.110666 35476988 PMC9119128

[14] Khalilgharibi N, Mao Y. To form and function: on the role of basement membrane mechanics in tissue development, homeostasis and disease. Open Biol. 2021;11(2):200360. doi: 10.1098/rsob.200360 33593159 PMC8061686

[15] Herrera-Perez RM, Cupo C, Allan C, Lin AJ, Kasza KE. Using optogenetics to link myosin patterns to contractile cell behaviors during convergent extension. Biophys J. 2021. doi: 10.1016/j.bpj.2021.06.041 34293302 PMC8516680

[16] Mira-Osuna M, Borgne RL. Assembly, dynamics and remodeling of epithelial cell junctions throughout development. Development. 2024;151(1):dev201086. doi: 10.1242/dev.201086 38205947

[17] Honda H. Description of cellular patterns by Dirichlet domains: the two-dimensional case. J Theor Biol. 1978;72(3):523–43. doi: 10.1016/0022-5193(78)90315-6 672241

[18] Honda H, Eguchi G. How much does the cell boundary contract in a monolayered cell sheet? J Theor Biol. 1980;84(3):575–88. doi: 10.1016/s0022-5193(80)80021-x 7431941

[19] Honda H. Geometrical models for cells in tissues. Int Rev Cytol. 1983;81:191–248. doi: 10.1016/s0074-7696(08)62339-6 6347934

[20] Nagai T, Kawasaki K, Nakamura K. Vertex dynamics of two-dimensional cellular patterns. J Phys Soc Jpn. 1988;57(7):2221–4. doi: 10.1143/jpsj.57.2221

[21] Weliky M, Oster G. The mechanical basis of cell rearrangement. I. Epithelial morphogenesis during Fundulus epiboly. Development. 1990;109(2):373–86. doi: 10.1242/dev.109.2.373 2401201

[22] Nagai T, Honda H. A dynamic cell model for the formation of epithelial tissues. Philos Magaz B. 2001;81(7):699–719. doi: 10.1080/13642810108205772

[23] Farhadifar R, Röper J-C, Aigouy B, Eaton S, Jülicher F. The influence of cell mechanics, cell-cell interactions, and proliferation on epithelial packing. Curr Biol. 2007;17(24):2095–104. doi: 10.1016/j.cub.2007.11.049 18082406

[24] Honda H, Tanemura M, Nagai T. A three-dimensional vertex dynamics cell model of space-filling polyhedra simulating cell behavior in a cell aggregate. J Theor Biol. 2004;226(4):439–53. doi: 10.1016/j.jtbi.2003.10.001 14759650

[25] Honda H, Nagai T, Tanemura M. Two different mechanisms of planar cell intercalation leading to tissue elongation. Developm Dyn. 2008;237(7):1826–36. doi: 10.1002/dvdy.21609 18570249

[26] Okuda S, Inoue Y, Eiraku M, Adachi T, Sasai Y. Vertex dynamics simulations of viscosity-dependent deformation during tissue morphogenesis. Biomech Model Mechanobiol. 2014;14(2):413–25. doi: 10.1007/s10237-014-0613-5 25227116

[27] Alt S, Ganguly P, Salbreux G. Vertex models: from cell mechanics to tissue morphogenesis. Philos Trans R Soc Lond B Biol Sci. 2017;372(1720):20150520. doi: 10.1098/rstb.2015.0520 28348254 PMC5379026

[28] Fletcher AG, Osterfield M, Baker RE, Shvartsman SY. Vertex models of epithelial morphogenesis. Biophys J. 2014;106(11):2291–304. doi: 10.1016/j.bpj.2013.11.4498 24896108 PMC4052277

[29] Bi D, Lopez J, Schwarz JM, Manning ML. A density-independent rigidity transition in biological tissues. Nat Phys. 2015;11(12):1074–9.

[30] Lawson-Keister E, Manning ML. Jamming and arrest of cell motion in biological tissues. Curr Opin Cell Biol. 2021;72:146–55. doi: 10.1016/j.ceb.2021.07.011 34461581

[31] Hannezo E, Heisenberg C-P. Rigidity transitions in development and disease. Trends Cell Biol. 2022;32(5):433–44. doi: 10.1016/j.tcb.2021.12.006 35058104

[32] Lan H, Wang Q, Fernandez-Gonzalez R, Feng JJ. A biomechanical model for cell polarization and intercalation during Drosophila germband extension. Phys Biol. 2015;12(5):056011. doi: 10.1088/1478-3975/12/5/056011 26356256

[33] Tetley RJ, Blanchard GB, Fletcher AG, Adams RJ, Sanson B. Unipolar distributions of junctional Myosin II identify cell stripe boundaries that drive cell intercalation throughout Drosophila axis extension. eLife. 2016;5:e12094. doi: 10.7554/elife.12094 27183005 PMC4915814

[34] Noll N, Mani M, Heemskerk I, Streichan SJ, Shraiman BI. Active tension network model suggests an exotic mechanical state realized in epithelial tissues. Nat Phys. 2017;13(12):1221–6. doi: 10.1038/nphys4219 30687408 PMC6344062

[35] Durney CH, Harris TJC, Feng JJ. Dynamics of PAR proteins explain the oscillation and ratcheting mechanisms in dorsal closure. Biophys J. 2018;115 11:2230–41. doi: 10.1016/j.bpj.2018.10.014 30446158 PMC6289075

[36] Tah I, Haertter D, Crawford JM, Kiehart DP, Schmidt CF, Liu AJ. Minimal vertex model explains how the amnioserosa avoids fluidization during Drosophila dorsal closure. arXiv preprint. 2023. https://arxiv.org/abs/2312.12926 10.1073/pnas.2322732121 PMC11725931 39793057

[37] Tetley RJ, Staddon MF, Heller D, Hoppe A, Banerjee S, Mao Y. Tissue fluidity promotes epithelial wound healing. Nat Phys. 2019;15(11):1195–1203. doi: 10.1038/s41567-019-0618-1 31700525 PMC6837871

[38] Honda H, Nagai T. Mathematical models of cell-based morphogenesis. Singapore: Springer; 2022.

[39] Staple DB, Farhadifar R, Röper JC, Aigouy B, Eaton S, Jülicher F. Mechanics and remodelling of cell packings in epithelia. Eur Phys J E Soft Matter. 2010;33(2):117–27. doi: 10.1140/epje/i2010-10677-0 21082210

[40] Popovic M, Druelle V, Dye NA, Jülicher F, Wyart M. Inferring the flow properties of epithelial tissues from their geometry. New J Phys. 2021;23(3):033004. doi: 10.1088/1367-2630/abcbc7

[41] Ogita G, Kondo T, Ikawa K, Uemura T, Ishihara S, Sugimura K. Image-based parameter inference for epithelial mechanics. PLOS Comput Biol. 2022;18(6):e1010209. doi: 10.1371/journal.pcbi.1010209 35737656 PMC9223404

[42] Damavandi OK, Arzash S, Lawson-Keister E, Manning ML. Universality in the mechanical behavior of vertex models for biological tissues. bioRxiv. 2022. doi: 10.1101/2022.06.01.494406

[43] Martin AC. Pulsation and stabilization: contractile forces that underlie morphogenesis. Develop Biol. 2010;341(1):114–25. doi: 10.1016/j.ydbio.2009.10.031 19874815

[44] Li X, Das A, Bi D. Mechanical heterogeneity in tissues promotes rigidity and controls cellular invasion. Phys Rev Lett. 2019;123(5):058101. doi: 10.1103/physrevlett.123.058101 31491312

[45] Siang LC, Fernandez-Gonzalez R, Feng JJ. Modeling cell intercalation during Drosophila germband extension. Phys Biol. 2018;15(6):066008. doi: 10.1088/1478-3975/aad865 30080681

[46] Sussman DM. Interplay of curvature and rigidity in shape-based models of confluent tissue. Phys Rev Res. 2020;2(2):023417. 10.1103/physrevresearch.2.023417

[47] Merkel M, Baumgarten K, Tighe BP, Manning ML. A minimal-length approach unifies rigidity in underconstrained materials. Proc Natl Acad Sci U S A. 2019;116(14):6560–8. doi: 10.1073/pnas.1815436116 30894489 PMC6452732

[48] Odell GM, Oster G, Alberch P, Burnside B. The mechanical basis of morphogenesis. Develop Biol. 1981;85(2):446–62. doi: 10.1016/0012-1606(81)90276-1 7196351

[49] Ishihara S, Sugimura K. Bayesian inference of force dynamics during morphogenesis. J Theor Biol. 2012;313:201–11. doi: 10.1016/j.jtbi.2012.08.017 22939902

[50] Ishihara S, Sugimura K, Cox SJ, Bonnet I, Bellaïche Y, Graner F. Comparative study of non-invasive force and stress inference methods in tissue. Eur Phys J E. 2013;36(4). doi: 10.1140/epje/i2013-13045-8 23615875

[51] Roffay C, Chan CJ, Guirao B, Hiiragi T, Graner F. Inferring cell junction tension and pressure from cell geometry. Development. 2021;148(18):dev192773. doi: 10.1242/dev.192773 33712442

[52] Kursawe J, Baker RE, Fletcher AG. Impact of implementation choices on quantitative predictions of cell-based computational models. J Comput Phys. 2017;345:752–67. doi: 10.1016/j.jcp.2017.05.048

[53] Park JA, Kim JH, Bi D, Mitchel JA, Qazvini NT, Tantisira K, et al. Unjamming and cell shape in the asthmatic airway epithelium. Nat Mater. 2015;14(10):1040–8. doi: 10.1038/nmat4357 26237129 PMC4666305

[54] Jain A, Ulman V, Mukherjee A, Prakash M, Cuenca MB, Pimpale LG, et al. Regionalized tissue fluidization is required for epithelial gap closure during insect gastrulation. Nat Commun. 2020;11(1). 33154375 10.1038/s41467-020-19356-x PMC7645651

[55] Rauzi M, Verant P, Lecuit T, Lenne PF. Nature and anisotropy of cortical forces orienting Drosophila tissue morphogenesis. Nat Cell Biol. 2008;10(12):1401–10. doi: 10.1038/ncb1798 18978783

[56] Merkel M, Manning ML. A geometrically controlled rigidity transition in a model for confluent 3D tissues. New J Phys. 2018;20(2):022002. doi: 10.1088/1367-2630/aaaa13

[57] Yan VT, Narayanan A, Wiegand T, Jülicher F, Grill SW. A condensate dynamic instability orchestrates actomyosin cortex activation. Nature. 2022;609(7927):597–604. doi: 10.1038/s41586-022-05084-3 35978196 PMC9477739

[58] Thiyagarajan S, Wang S, Chew TG, Huang J, Kumar L, Balasubramanian MK, et al. Myosin turnover controls actomyosin contractile instability. Proc Natl Acad Sci. 2022;119(43):e2211431119. doi: 10.1073/pnas.2211431119 36264833 PMC9618044

[59] Sánchez-Gutiérrez D, Tozluoglu M, Barry JD, Pascual A, Mao Y, Escudero LM. Fundamental physical cellular constraints drive self-organization of tissues. EMBO J. 2016;35:77–88. doi: 10.15252/embj.201592374 26598531 PMC4718000

[60] Curran S, Strandkvist C, Bathmann J, de Gennes M, Kabla A, Salbreux G, et al. Myosin II controls junction fluctuations to guide epithelial tissue ordering. Dev Cell. 2017;43(4):480–92.e6. doi: 10.1016/j.devcel.2017.09.018 29107560 PMC5703647

[61] Duclut C, Paijmans J, Inamdar MM, Modes CD, Jülicher F. Nonlinear rheology of cellular networks. Cells Dev. 2021;168:203746. doi: 10.1016/j.cdev.2021.203746 34592496

[62] Rozman J, Krajnc M, Ziherl P. Morphologies of compressed active epithelial monolayers. Eur Phys J E Soft Matter. 2021;44(7):99. doi: 10.1140/epje/s10189-021-00094-x 34287727

[63] Devany J, Sussman DM, Yamamoto T, Manning ML, Gardel ML. Cell cycle-dependent active stress drives epithelia remodeling. Proc Natl Acad Sci U S A. 2021;118:e1917853118. doi: 10.1073/pnas.1917853118 33649197 PMC7958291

[64] Yamamoto T, Sussman DM, Shibata T, Manning ML. Non-monotonic fluidization generated by fluctuating edge tensions in confluent tissues. Soft Matter. 2022;18(11):2168–75. doi: 10.1039/d0sm01559h 35212696

[65] Perez-Verdugo F, Banerjee S. Tension Remodeling Regulates Topological Transitions in Epithelial Tissues. PRX Life. 2023;1(2):023006. doi: 10.1103/prxlife.1.023006 PMC11500814 39450340

[66] Erdemci-Tandogan G, Manning ML. Effect of cellular rearrangement time delays on the rheology of vertex models for confluent tissues. PLOS Comput Biol. 2021;17. doi: 10.1101/2021.02.13.431087 PMC8211246 34097706

[67] Sato K, Hiraiwa T, Maekawa E, Isomura A, Shibata T, Kuranaga E. Left-right asymmetric cell intercalation drives directional collective cell movement in epithelial morphogenesis. Nat Commun. 2015;6():10074. doi: 10.1038/ncomms10074 26656655 PMC4682055

[68] Sato K, Hiraiwa T, Shibata T. Cell chirality induces collective cell migration in epithelial sheets. Phys Rev Lett. 2015;115(18):188102. doi: 10.1103/physrevlett.115.188102 26565500

[69] Hiraiwa T, Kuranaga E, Shibata T. Wave propagation of junctional remodeling in collective cell movement of epithelial tissue: numerical simulation study. Front Cell Dev Biol. 2017;5:66. doi: 10.3389/fcell.2017.00066 28770197 PMC5516087

[70] Jacinto A, Wood W, Woolner S, Hiley C, Turner L, Wilson C, et al. Dynamic analysis of actin cable function during drosophila dorsal closure. Curr Biol. 2002;12(14):1245–50. doi: 10.1016/s0960-9822(02)00955-7 12176336

[71] Hutson MS, Tokutake Y, Chang MS, Bloor JW, Venakides S, Kiehart DP, et al. Forces for morphogenesis investigated with laser microsurgery and quantitative modeling. Science. 2003;300:145–9. doi: 10.1126/science.1079552 12574496

[72] Solon J, Kaya-Copur A, Colombelli J, Brunner D. Pulsed forces timed by a ratchet-like mechanism drive directed tissue movement during dorsal closure. Cell. 2009;137:1331–42. doi: 10.1016/j.cell.2009.03.050 19563762

[73] Benton MA, Akam M, Pavlopoulos A. Cell and tissue dynamics during Tribolium embryogenesis revealed by versatile fluorescence labeling approaches. Development. 2013;140:3210–20. doi: 10.1242/dev.096271 23861059 PMC3930475

[74] Pasakarnis L, Frei E, Caussinus E, Affolter M, Brunner D. Amnioserosa cell constriction but not epidermal actin cable tension autonomously drives dorsal closure. Nat Cell Biol. 2016;18:1161–72. doi: 10.1038/ncb3420 27749821

[75] Lo Vecchio S, Pertz O, Szopos M, Navoret L, Riveline D. Spontaneous rotations in epithelia as an interplay between cell polarity and boundaries. Nat Phys. 2024;20(2):322–331. doi: 10.1038/s41567-023-02295-x

[76] Landsberg KP, Farhadifar R, Ranft J, Umetsu D, Widmann TJ, Bittig T, et al. Increased cell bond tension governs cell sorting at the Drosophila anteroposterior compartment boundary. Curr Biol. 2009;19(22):1950–1955. doi: 10.1016/j.cub.2009.10.021 19879142

[77] Murisic N, Hakim V, Kevrekidis IG, Shvartsman SY, Audoly B. From discrete to continuum models of three-dimensional deformations in epithelial sheets. Biophys J. 2015;109(1):154–63. doi: 10.1016/j.bpj.2015.05.019 26153712 PMC4571022

[78] Durney CH, Feng JJ. A three-dimensional vertex model for Drosophila salivary gland invagination. Phys Biol. 2021;18(4):046005. doi: 10.1088/1478-3975/abfa69 33882465

[79] Perez-Verdugo F, Reig G, Cerda M, Concha ML, Soto R. Geometrical characterization of active contraction pulses in epithelial cells using the two-dimensional vertex model. J Roy Soc Interface. 2022;19(186):20210851. doi: 10.1098/rsif.2021.0851 PMC8790349 35078339

[80] Canela-Xandri O, Sagués F, Casademunt J, Buceta J. Dynamics and mechanical stability of the developing dorsoventral organizer of the wing imaginal disc. PLOS Comput Biol. 2011;7(9):e1002153. doi: 10.1371/journal.pcbi.1002153 21980267 PMC3182857

[81] Wang Q, Feng J, Pismen L. A cell-level biomechanical model of Drosophila dorsal closure. Biophys J. 2012;103(11):2265–74. doi: 10.1016/j.bpj.2012.09.036 23283225 PMC3514517

[82] López-Gay JM, Nunley H, Spencer M, di Pietro F, Guirao B, Bosveld F, et al. Apical stress fibers enable a scaling between cell mechanical response and area in epithelial tissue. Science. 2020;370(6514):eabb2169. doi: 10.1126/science.abb2169 33060329

[83] Inaki M, Hatori R, Nakazawa N, Okumura T, Ishibashi T, Kikuta J, et al. Chiral cell sliding drives left-right asymmetric organ twisting. eLife. 2018;7:e32506. doi: 10.7554/elife.32506 29891026 PMC5997448

[84] Honda H. Left-handed cardiac looping by cell chirality is mediated by position-specific convergent extensions. Biophys J. 2021;120(23):5371–83. doi: 10.1016/j.bpj.2021.10.025 34695385 PMC8715179

[85] Martin AC, Kaschube M, Wieschaus EF. Pulsed contractions of an actin–myosin network drive apical constriction. Nature. 2009;457(7228):495–9. doi: 10.1038/nature07522 19029882 PMC2822715

[86] Blanchard GB, Étienne J, Gorfinkiel N. From pulsatile apicomedial contractility to effective epithelial mechanics. Curr Opin Genet Dev. 2018;51:78–87. doi: 10.1016/j.gde.2018.07.004 30077073

[87] Chuai M, Weijer CJ. Regulation of cell migration during chick gastrulation. Curr Opin Genet Develop. 2009;19(4):343–9. doi: 10.1016/j.gde.2009.06.007 19647425

[88] Ivanov AI, Hunt D, Utech M, Nusrat A, Parkos CA. Differential roles for actin polymerization and a myosin II motor in assembly of the epithelial apical junctional complex. Molecul Biol Cell. 2005;16(6):2636–50. doi: 10.1091/mbc.e05-01-0043 15800060 PMC1142412

[89] Klingner C, Cherian AV, Fels J, Diesinger PM, Aufschnaiter R, Maghelli N, et al. Isotropic actomyosin dynamics promote organization of the apical cell cortex in epithelial cells. J Cell Biol. 2014;207(1):107–21. doi: 10.1083/jcb.201402037 25313407 PMC4195824

[90] Finegan TM, Hervieux N, Nestor-Bergmann A, Fletcher AG, Blanchard GB, Sanson B. The tricellular vertex-specific adhesion molecule Sidekick facilitates polarised cell intercalation during Drosophila axis extension. PLOS Biol. 2019;17:e3000522. doi: 10.1371/journal.pbio.3000522 31805038 PMC6894751

[91] Yu HH, Zallen JA. Abl and Canoe/Afadin mediate mechanotransduction at tricellular junctions. Science. 2020;370(6520):eaba5528. doi: 10.1126/science.aba5528 33243859 PMC8559527

[92] Bosveld F, Bellaïche Y. Tricellular junctions. Curr Biol. 2020;30(6):R249–51. doi: 10.1016/j.cub.2020.01.029 32208143

[93] Higashi T, Chiba H. Molecular organization, regulation and function of tricellular junctions. Biochim Biophys Acta Biomembr. 2020;1862(2):183143. doi: 10.1016/j.bbamem.2019.183143 31812626

[94] Choi W, Acharya BR, Peyret G, Fardin M-A, Mège R-M, Ladoux B, et al. Remodeling the zonula adherens in response to tension and the role of afadin in this response. J Cell Biol. 2016;213(2):243–260. doi: 10.1083/jcb.201506115 27114502 PMC5084271

[95] Cho Y, Haraguchi D, Shigetomi K, Matsuzawa K, Uchida S, Ikenouchi J. Tricellulin secures the epithelial barrier at tricellular junctions by interacting with actomyosin. J Cell Biol. 2022;221(4):e202009037. doi: 10.1083/jcb.202009037 35148372 PMC8847807

[96] Barai A, Soleilhac M, Xi W, Lin SZ, Karnat M, Bazelli`eres E, et al. A multicellular actin star network underpins epithelial organization and connectivity. bioRxiv. 2024. doi: 10.1101/2024.07.26.605277 PMC12227619 40615382

[97] Fang C, Shao X, Tian Y, Chu Z, Lin Y. Size-dependent response of cells in epithelial tissue modulated by contractile stress fibers. Biophys J. 2023;122(7):1315–24. doi: 10.1016/j.bpj.2023.02.026 36809876 PMC10111366

[98] Tlili S, Yin J, Rupprecht JF, Mendieta-Serrano MA, Weissbart G, Verma N, et al. Shaping the zebrafish myotome by intertissue friction and active stress. Proc Natl Acad Sci. 2019;116(51):25430–9. doi: 10.1073/pnas.1900819116 31772022 PMC6925982

[99] Comelles J, SS S, Lu L, Le Maout E, Anvitha S, Salbreux G, et al. Epithelial colonies in vitro elongate through collective effects. eLife. 2021;10:e57730. doi: 10.7554/elife.57730 33393459 PMC7850623

[100] Lin SZ, Merkel M, Rupprecht JF. Implementation of cellular bulk stresses in vertex models of biological tissues. Eur Phys J E. 2022;45(1). doi: 10.1140/epje/s10189-021-00154-2 35038043

[101] Odell G, Oster G, Burnside B, Alberch P. A mechanical model for epithelial morphogenesis. J Math Biol. 1980; 9(3):291–5. doi: 10.1007/bf00276030 7190180

[102] Benham-Pyle BW, Pruitt BL, Nelson WJ. Mechanical strain induces E-cadherin-dependent Yap1 and *β*-catenin activation to drive cell cycle entry. Science. 2015;348:1024–7. doi: 10.1126/science.aaa4559 26023140 PMC4572847

[103] Acharya BR, Nestor-Bergmann A, Liang X, Gupta S, Duszyc K, Gauquelin E, et al. A Mechanosensitive RhoA pathway that protects epithelia against acute tensile stress. Dev Cell. 2018;47(4):439–452.e6. doi: 10.1016/j.devcel.2018.09.016 30318244

[104] Sumi A, Hayes P, D’Angelo A, Colombelli J, Salbreux G, Dierkes K, et al. Adherens junction length during tissue contraction is controlled by the mechanosensitive activity of actomyosin and junctional recycling. Dev Cell. 2018;47(4):453–63.e3. doi: 10.1016/j.devcel.2018.10.025 30458138 PMC6291457

[105] Duda M, Kirkland NJ, Khalilgharibi N, Tozluoglu M, Yuen AC, Carpi N, et al. Polarization of Myosin II refines tissue material properties to buffer mechanical stress. Developm Cell. 2019;48(2):245–260.e7. doi: 10.1016/j.devcel.2018.12.020 PMC6353629 30695698

[106] Andreu I, Falcones B, Hurst S, Chahare NR, Quiroga X, Roux A-L-L, et al. The force loading rate drives cell mechanosensing through both reinforcement and cytoskeletal softening. Nat Commun. 2021;12:4229. doi: 10.1038/s41467-021-24383-3 34244477 PMC8270983

[107] Lenne PF, Rupprecht JF, Viasnoff V. Cell junction mechanics beyond the bounds of adhesion and tension. Developm Cell. 2021;56(2):202–12. doi: 10.1016/j.devcel.2020.12.018 33453154

[108] Cavanaugh KE, Staddon MF, Munro E, Banerjee S, Gardel ML. RhoA mediates epithelial cell shape changes via mechanosensitive endocytosis. Developm Cell. 2020;52(2):152–66.e5. doi: 10.1016/j.devcel.2019.12.002 31883774 PMC7565439

[109] Gustafson HJ, Claussen N, De Renzis S, Streichan SJ. Patterned mechanical feedback establishes a global myosin gradient. Nat Commun. 2022;13(1):7050. doi: 10.1038/s41467-022-34518-9 36396633 PMC9672098

[110] Nishimura R, Kato K, Saida M, Kamei Y, Takeda M, Miyoshi H, et al. Appropriate tension sensitivity of *α*-catenin ensures rounding morphogenesis of epithelial spheroids. Cell Struct Funct. 2022;47(2):55–73. doi: 10.1247/csf.22014 35732428 PMC10511042

[111] Collinet C, Lecuit T. Programmed and self-organized flow of information during morphogenesis. Nat Rev Mol Cell Biol. 2021;22(4):245–65. doi: 10.1038/s41580-020-00318-6 33483696

[112] Okuda S, Takata N, Hasegawa Y, Kawada M, Inoue Y, Adachi T, et al. Strain-triggered mechanical feedback in self-organizing optic-cup morphogenesis. Sci Adv. 2018;4(11):eaau1354. doi: 10.1126/sciadv.aau1354 30474058 PMC6248953

[113] Staddon MF, Cavanaugh KE, Munro EM, Gardel ML, Banerjee S. Mechanosensitive junction remodeling promotes robust epithelial morphogenesis. Biophys J. 2019;117(9):1739–50. doi: 10.1016/j.bpj.2019.09.027 31635790 PMC6838884

[114] Cl´ement R, Dehapiot B, Collinet C, Lecuit T, Lenne PF. Viscoelastic dissipation stabilizes cell shape changes during tissue morphogenesis. Curr Biol. 2017;27(20):3132–42.e4. doi: 10.1016/j.cub.2017.09.005 28988857

[115] Wen FL, Kwan CW, Wang YC, Shibata T. Autonomous epithelial folding induced by an intracellular mechano–polarity feedback loop. PLOS Comput Biol. 2021;17(12):e1009614. doi: 10.1371/journal.pcbi.1009614 PMC8675927 34871312

[116] Armon S, Bull MS, Moriel A, Aharoni H, Prakash M. Modeling epithelial tissues as active-elastic sheets reproduce contraction pulses and predict rip resistance. Commun Phys. 2021;4:1–9. doi: 10.1038/s42005-021-00712-2

[117] Sknepnek R, Djafer-Cherif I, Chuai M, Weijer C, Henkes S. Generating active T1 transitions through mechanochemical feedback. eLife. 2023;12:e79862. doi: 10.7554/elife.79862 37039463 PMC10156166

[118] Tan TH, Amiri A, Seijo-Barandiaran I, Staddon MF, Materne A, Tomas S, et al. Emergent chirality in active solid rotation of pancreas spheres. PRX Life. 2024;2(3):033006. doi: 10.1103/prxlife.2.033006

[119] Cohen R, Taiber S, Loza O, Kasirer S, Woland S, Sprinzak D. Precise alternating cellular pattern in the inner ear by coordinated hopping intercalations and delaminations. Sci Adv. 2023;9(8):eadd2157. doi: 10.1126/sciadv.add2157 36812313 PMC12488031

[120] Yan L, Bi D. Multicellular rosettes drive fluid-solid transition in epithelial tissues. Phys Rev X. 2019;9:(1). doi: 10.1103/physrevx.9.011029

[121] Thomas EC, Hopyan S. Shape-driven confluent rigidity transition in curved biological tissues. Biophys J. 2023;122(21):4264–73. doi: 10.1016/j.bpj.2023.10.001 37803831 PMC10645569

[122] De Marzio M, Das A, Fredberg JJ, Bi D. Epithelial layer fluidization by curvature-induced unjamming. arXiv preprint 2023. https://arxiv.org/abs/2305.12667 10.1103/PhysRevLett.134.138402 PMC12088683 40250361

[123] Staddon MF, Modes CD. Curved edges in the vertex model increase tissue fluidity. arXiv preprint 2024. https://arxiv.org/abs/2410.06821

[124] Lee CT, Merkel M. Generic elasticity of thermal, under-constrained systems. arXiv preprint 2023. https://arxiv.org/abs/2304.07266

[125] Barton DL, Henkes S, Weijer CJ, Sknepnek R. Active vertex model for cell-resolution description of epithelial tissue mechanics. PLOS Comput Biol. 2017;13(6):e1005569. doi: 10.1371/journal.pcbi.1005569 PMC5493290 28665934

[126] Cislo DJ, Yang F, Qin H, Pavlopoulos A, Bowick MJ, Streichan SJ. Active cell divisions generate fourfold orientationally ordered phase in living tissue. Nat Phys. 2023;19(8):1201–10. doi: 10.1038/s41567-023-02025-3 37786880 PMC10545346

[127] Guerrero P, Perez-Carrasco R. Choice of friction coefficient deeply affects tissue behaviour in stochastic epithelial vertex models. Philos Trans R Soc Lond B Biol Sci. 2024;379(1900):20230051. doi: 10.1098/rstb.2023.0051 38432320 PMC10909505

[128] Staple D. Understanding mechanics and polarity in two-dimensional tissues [Ph.D. thesis]. Technische Universität Dresden; 2012.

[129] Tong S, Sknepnek R, Kosmrlj A. Linear viscoelastic response of the vertex model with internal and external dissipation: Normal modes analysis. Phys Rev Res. 2023;5(1):013143. doi: 10.1103/physrevresearch.5.013143

[130] Rozman J, CKV S, Yeomans JM, Sknepnek R. From dry to wet vertex model dynamics: generating sustained flows. arXiv preprint 2023. https://arxiv.org/abs/2312.11756

[131] Fu C, Dilasser F, Lin SZ, Karnat M, Arora A, Rajendiran H, et al. Regulation of intercellular viscosity by E-cadherin-dependent phosphorylation of EGFR in collective cell migration. Proc Natl Acad Sci. 2024;121(37). doi: 10.1073/pnas.2405560121 PMC11406304 39231206

[132] Brodland GW, Conte V, Cranston PG, Veldhuis J, Narasimhan S, Hutson MS, et al. Video force microscopy reveals the mechanics of ventral furrow invagination in Drosophila. Proc Natl Acad Sci U S A. 2010;107(51):22111–6. doi: 10.1073/pnas.1006591107 21127270 PMC3009801

[133] Chiou KK, Hufnagel L, Shraiman BI. Mechanical stress inference for two dimensional cell arrays. PLOS Comput Biol. 2012;8(5):e1002512. doi: 10.1371/journal.pcbi.1002512 PMC3355066 22615550

[134] Noll N, Streichan SJ, Shraiman BI. Variational method for image-based inference of internal stress in epithelial tissues. Phys Rev X. 2020;10:011072. doi: 10.1103/PhysRevX.10.011072 33767909 PMC7989596

[135] Moshe M, Bowick MJ, Marchetti MC. Geometric frustration and solid-solid transitions in model 2D tissue. Phys Rev Lett. 2018;120(26):268105. doi: 10.1103/physrevlett.120.268105 30004729

[136] Tong S, Singh NK, Sknepnek R, Koˇsmrlj A. Linear viscoelastic properties of the vertex model for epithelial tissues. PLOS Comput Biol. 2022;18(5):e1010135. doi: 10.1371/journal.pcbi.1010135 PMC9159552 35587514

[137] Krajnc M, Dasgupta S, Ziherl P, Prost J. Fluidization of epithelial sheets by active cell rearrangements. Phys Rev E. 2018;98(2):022409. doi: 10.1103/physreve.98.022409 30253464

[138] Fielding SM, Cochran JO, Huang J, Bi D, Marchetti MC. Constitutive model for the rheology of biological tissue. Phys Rev E. 2023;108(4):L042602. doi: 10.1103/PhysRevE.108.L042602 37978678

[139] Ishihara S, Marcq P, Sugimura K. From cells to tissue: a continuum model of epithelial mechanics. Phys Rev E. 2017;96(2):022418. doi: 10.1103/physreve.96.022418 28950595

[140] Triguero-Platero G, Ziebert F, Bonilla LL. Coarse-graining the vertex model and its response to shear. Phys Rev E. 2023;108(4):044118. doi: 10.1103/physreve.108.044118 37978645

[141] Grossman D, Joanny J-F. Instabilities and geometry of growing tissues. Phys Rev Lett. 2022;129(4):048102. doi: 10.1103/PhysRevLett.129.048102 35938996

[142] Bi D, Yang X, Marchetti MC, Manning ML. Motility-driven glass and jamming transitions in biological tissues. Phys Rev X. 2016;6(2):021011. doi: 10.1103/PhysRevX.6.021011 28966874 PMC5619672

[143] Sussman DM, Merkel M. No unjamming transition in a Voronoi model of biological tissue. Soft Matter. 2018;14(17):3397–403. doi: 10.1039/c7sm02127e 29667689

[144] Lawson-Keister E, Zhang T, Nazari F, Fagotto F, Manning ML. Differences in boundary behavior in the 3D vertex and Voronoi models. PLOS Comput Biol. 2024;20(1):e1011724. doi: 10.1371/journal.pcbi.1011724 38181065 PMC10796063

[145] Gómez-Gálvez P, Vicente-Munuera P, Tagua A, Forja C, Castro AM, Letrán M, et al. Scutoids are a geometrical solution to three-dimensional packing of epithelia. Nat Commun. 2018;9(1):2960. doi: 10.1038/s41467-018-05376-1 30054479 PMC6063940

[146] Gomez-Galvez P, Vicente-Munuera P, Anbari S, Tagua A, Gordillo-Vazquez C, Andres-San Roman JA, et al. A quantitative biophysical principle to explain the 3D cellular connectivity in curved epithelia. Cell Syst. 2022;13(8):631–43.e8. doi: 10.1016/j.cels.2022.06.003 35835108

[147] Gomez-Galvez P, Vicente-Munuera P, Anbari S, Buceta J, Escudero LM. The complex three-dimensional organization of epithelial tissues. Development. 2021;148(1). doi: 10.1242/dev.195669 33408064

[148] Ishimoto Y, Morishita Y. Bubbly vertex dynamics: a dynamical and geometrical model for epithelial tissues with curved cell shapes. Phys Rev E. 2014;90(5):052711. doi: 10.1103/physreve.90.052711 25493820

[149] Schaumann EN, Staddon MF, Gardel ML, Banerjee S. Force localization modes in dynamic epithelial colonies. Mol Biol Cell. 2018;29(23):2835–47. doi: 10.1091/mbc.e18-05-0336 30207837 PMC6249864

[150] Kim K, Schwarz JM, Ben Amar M. A two-dimensional vertex model for curvy cell-cell interfaces at the subcellular scale. J Roy Soc Interface. 2024;21(217):20240193. doi: 10.1098/rsif.2024.0193 39192725 PMC11407580

[151] Boromand A, Signoriello A, Ye F, O’Hern CS, Shattuck MD. Jamming of deformable polygons. Phys Rev Lett. 2018;121(24):248003. doi: 10.1103/physrevlett.121.248003 30608748

[152] Nestor-Bergmann A, Blanchard GB, Hervieux N, Fletcher AG, Étienne J, Sanson B. Adhesion-regulated junction slippage controls cell intercalation dynamics in an Apposed-Cortex Adhesion Model. PLoS Comput Biol. 2022;18:e1009812. doi: 10.1371/journal.pcbi.1009812 35089922 PMC8887740

[153] Mitchel JA, Das A, O’Sullivan MJ, Stancil IT, DeCamp SJ, Koehler S, et al. In primary airway epithelial cells, the unjamming transition is distinct from the epithelial-to-mesenchymal transition. Nat Commun. 2020;11(1). doi: 10.1038/s41467-020-18841-7 PMC7542457 33028821

[154] Huang J, Levine H, Bi D. Bridging the gap between collective motility and epithelial-mesenchymal transitions through the active finite voronoi model. Soft Matter. 2023;19(48):9389–98. doi: 10.1039/d3sm00327b 37795526 PMC10843280

[155] Miotto M, Ruocco G, Paoluzzi M. Non-equilibrium phase transitions in hybrid Voronoi models of cell colonies. arXiv prerpint 2024. https://arxiv.org/abs/2411.08012 10.1039/d6sm00238b 42385690

[156] Nonomura M. Study on multicellular systems using a phase field model. PLOS ONE. 2012;7(4):e33501. doi: 10.1371/journal.pone.0033501 22539943 PMC3335162

[157] Loewe B, Chiang M, Marenduzzo D, Marchetti MC. Solid-liquid transition of deformable and overlapping active particles. Phys Rev Lett. 2020;125(3):038003. doi: 10.1103/physrevlett.125.038003 32745423

[158] Jain HP, Voigt A, Angheluta L. Robust statistical properties of T1 transitions in a multi-phase field model of cell monolayers. Sci Rep. 2023;13(1):10096. doi: 10.1038/s41598-023-37064-6 37344548 PMC10284850

[159] Kupferman R, Maman B, Moshe M. Continuum mechanics of a cellular tissue model. J Mech Phys Solids. 2020;143:104085. doi: 10.1016/j.jmps.2020.104085

[160] Torres-Sánchez A, Kerr Winter M, Salbreux G. Interacting active surfaces: a model for three-dimensional cell aggregates. PLOS Comput Biol. 2022;18(12):e1010762. doi: 10.1371/journal.pcbi.1010762 36525467 PMC9803321

[161] Merks RMH, Glazier JA. A cell-centered approach to developmental biology. Phys A: Statist Mech Appl. 2005;352(1):113–30. doi: 10.1016/j.physa.2004.12.028

[162] Chiang M, Marenduzzo D. Glass transitions in the cellular Potts model. EPL. 2016;116(2):28009. doi: 10.1209/0295-5075/116/28009

[163] Sadhukhan S, Nandi SK. Theory and simulation for equilibrium glassy dynamics in cellular Potts model of confluent biological tissue. Phys Rev E. 2021;103(6):062403. doi: 10.1103/physreve.103.062403 34271700

[164] Nemati H, de Graaf J. The cellular Potts model on disordered lattices. Soft Matter. 2024;20(42):8337–52. doi: 10.1039/d4sm00445k 39283268 PMC11404401

[165] Brakke KA. The surface evolver. Exp Math. 1992;1(2):141–65. doi: 10.1080/10586458.1992.10504253

[166] Mirams GR, Arthurs CJ, Bernabeu MO, Bordas R, Cooper J, Corrias A, et al. Chaste: an open source C++ library for computational physiology and biology. PLOS Comput Biol. 2013;9(3):e1002970. doi: 10.1371/journal.pcbi.1002970 23516352 PMC3597547

[167] Fletcher AG, Osborne JM, Maini PK, Gavaghan DJ. Implementing vertex dynamics models of cell populations in biology within a consistent computational framework. Prog Biophys Molecul Biol. 2013;113(2):299–326. doi: 10.1016/j.pbiomolbio.2013.09.003 24120733

[168] Sussman DM. cellGPU: Massively parallel simulations of dynamic vertex models. Comput Phys Commun. 2017;219:400–6. doi: 10.1016/j.cpc.2017.06.001

[169] Canela-Xandri O, Anbari S, Buceta J. TiFoSi: an efficient tool for mechanobiology simulations of epithelia. Bioinformatics. 2020;36(16):4525–6. doi: 10.1093/bioinformatics/btaa592 32589697

[170] Theis S, Suzanne M, Gay G. Tyssue: an epithelium simulation library. J Open Source Softw. 2021;6(62):2973. doi: 10.21105/joss.02973

[171] Sego TJ, Comlekoglu T, Peirce SM, Desimone DW, Glazier JA. General, open-source vertex modeling in biological applications using Tissue Forge. Sci Rep. 2023;13(1):17886. doi: 10.1038/s41598-023-45127-x 37857673 PMC10587242

[172] Sarkar T, Krajnc M. Graph topological transformations in space-filling cell aggregates. PLOS Comput Biol. 2024;20(5):e1012089. doi: 10.1371/journal.pcbi.1012089 38743660 PMC11093388

